# Supplementary material for: Genome-wide association study of pigmentary traits (skin and iris color) in individuals of East Asian ancestry
Source: PeerJ. 2017 Nov 2;5:e3951. doi: 10.7717/peerj.3951 (PMC5671666; doi:10.7717/peerj.3951)
Supplement: Figure S6 — All these regions harbour multiple markers showing suggestive significance and good imputation scores (e.g., score info > 0.8). [file peerj-05-3951-s006.pdf]

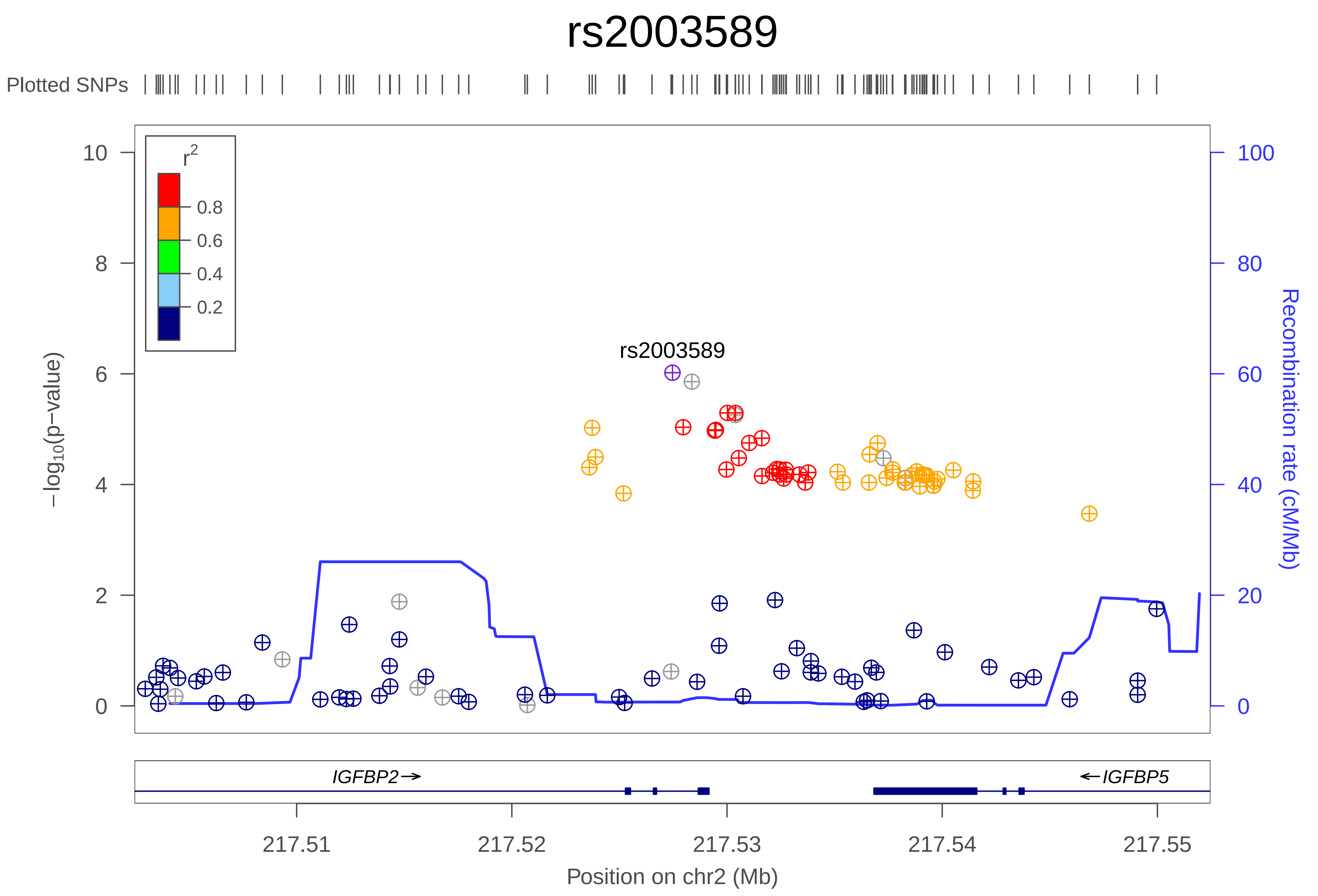
rs2003589
Plotted SNPs
r2
0.8
0.6
0.4
0.2
rs2003589
10
8
6
4
2
0
−log10(p−value)
100
80
60
40
20
0
Recombination rate (cM/Mb)
IGFBP2→
←IGFBP5
217.51
217.52
217.53
217.54
217.55
Position on chr2 (Mb)

date:	Wed Oct 19 17:08:09 2016
build:	hg19
display range:	chr2:217502465−217552465 [217502465−217552465]
hilite range:	0 − 0 [0 − 0]
reference SNP:	chr2:217527465
number of SNPs plotted:	120
min P.value:	9.54E−7 [chr2:217527465]
max P.value:	9.64E−1 [chr2:217520720]

annotation key

framestop	○
splice	□
nonsyn	◇
coding	△
utr	▽
tfbscons	×
mcs44placental	⊠
no annotation	✳
none	⊕

Make more plots at http://csg.sph.umich.edu/locuszoom/

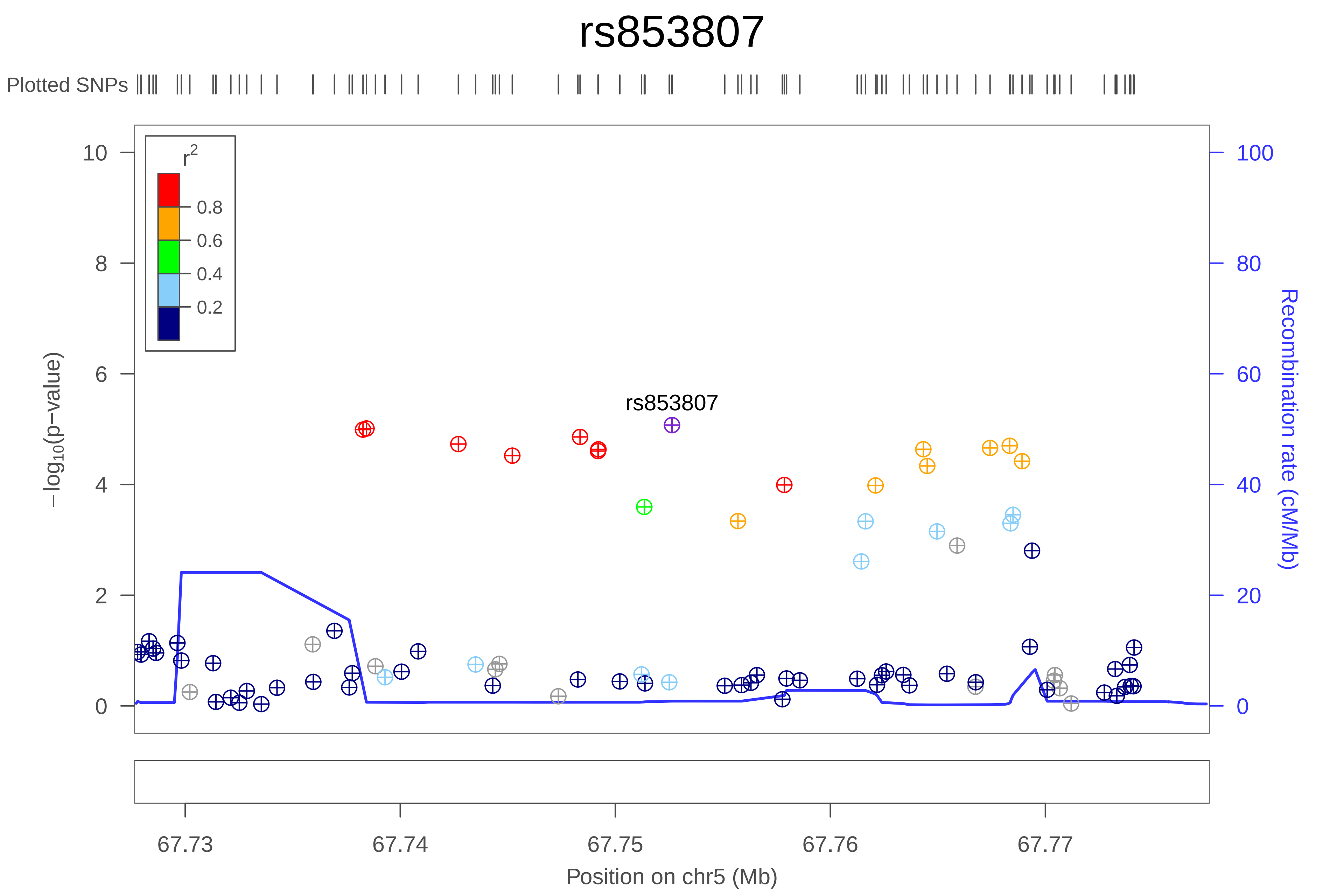
rs853807
Plotted SNPs
r2
0.8
0.6
0.4
0.2
rs853807
−log10(p−value)
Recombination rate (cM/Mb)
0
2
4
6
8
10
0
20
40
60
80
100
67.73
67.74
67.75
67.76
67.77
Position on chr5 (Mb)

date:	Wed Oct 19 17:12:58 2016
build:	hg19
display range:	chr5:67727638−67777638 [67727638−67777638]
hilite range:	0 − 0 [0 − 0]
reference SNP:	chr5:67752638
number of SNPs plotted:	89
min P.value:	8.45E−6 [chr5:67752638]
max P.value:	9.27E−1 [chr5:67733541]

annotation key

framestop	○
splice	□
nonsyn	◇
coding	△
utr	▽
tfbscons	×
mcs44placental	⊠
no annotation	✳
none	⊕

Make more plots at http://csg.sph.umich.edu/locuszoom/

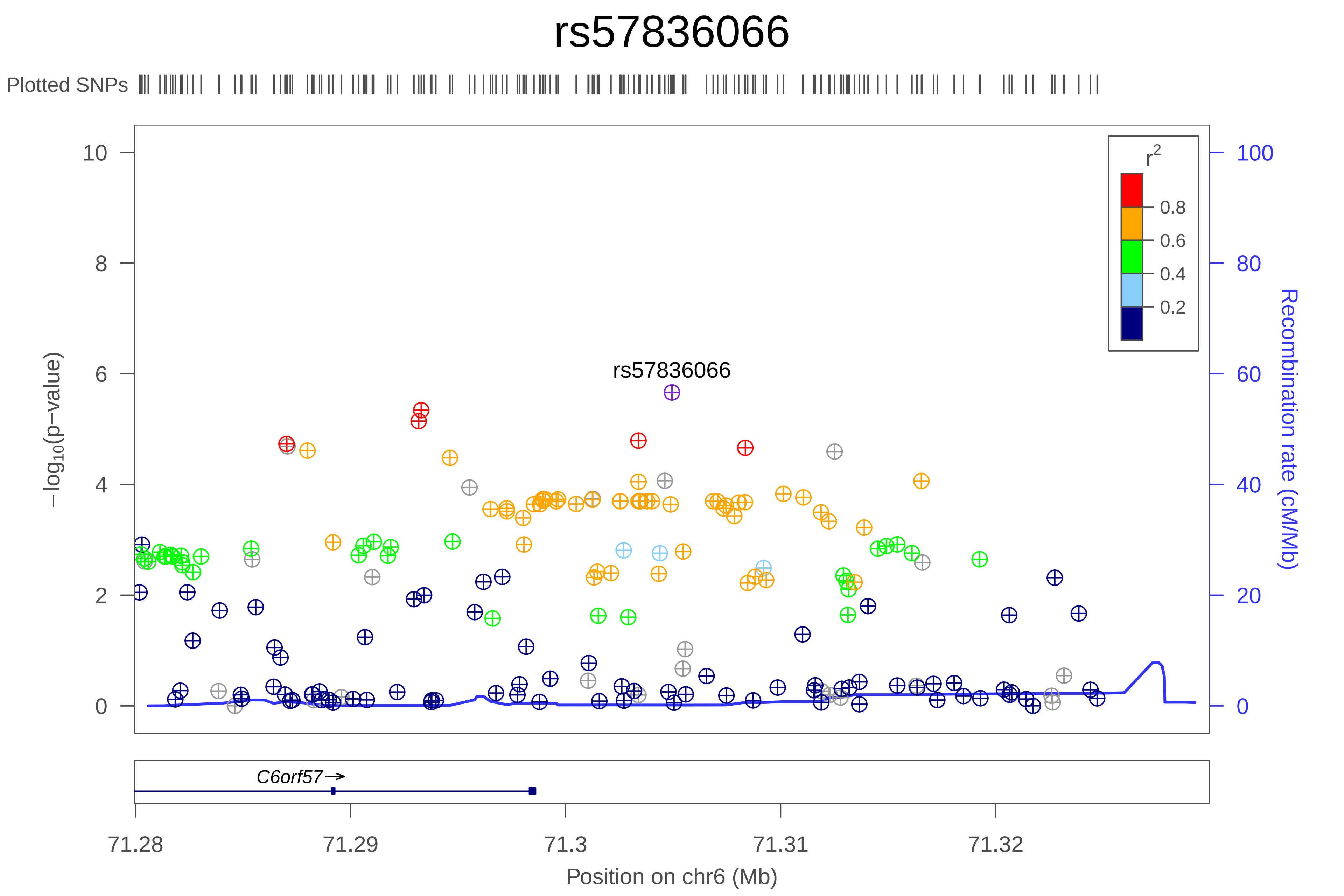

rs57836066
Plotted SNPs
−log10(p−value)
Recombination rate (cM/Mb)
r2
0.8
0.6
0.4
0.2
rs57836066
C6orf57→
71.28
71.29
71.3
71.31
71.32
Position on chr6 (Mb)
0
2
4
6
8
10
0
20
40
60
80
100

date:	Wed Oct 19 17:35:45 2016
build:	hg19
display range:	chr6:71279950−71329950 [71279950−71329950]
hilite range:	0 − 0 [0 − 0]
reference SNP:	chr6:71304950
number of SNPs plotted:	192
min P.value:	2.17E−6 [chr6:71304950]
max P.value:	9.98E−1 [chr6:71284625]

annotation key

framestop	○
splice	□
nonsyn	◇
coding	△
utr	▽
tfbscons	×
mcs44placental	⊠
no annotation	✳
none	⊕

Make more plots at http://csg.sph.umich.edu/locuszoom/

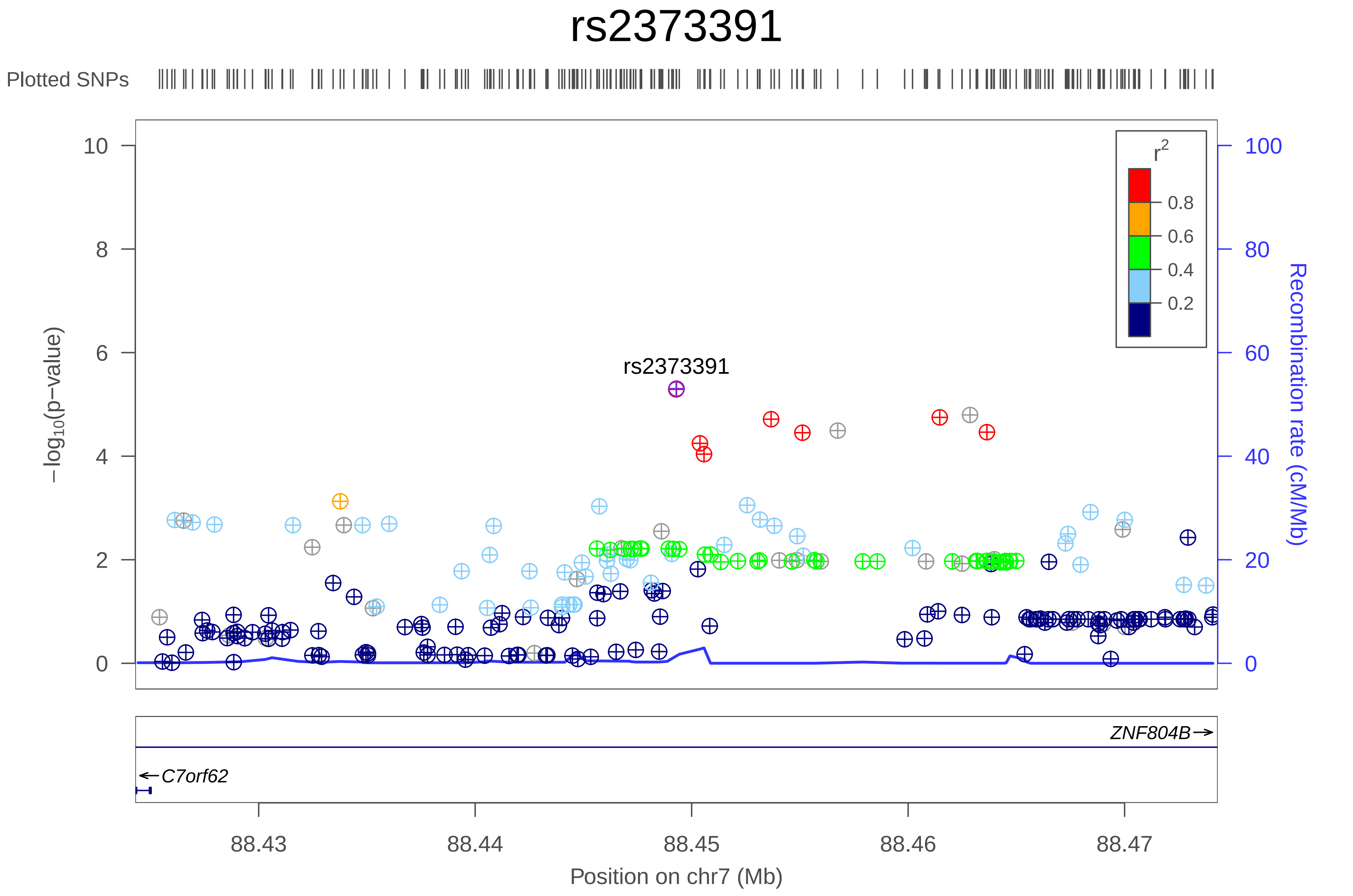
rs2373391
Plotted SNPs
10
8
6
4
2
0
−log10(p−value)
100
80
60
40
20
0
Recombination rate (cM/Mb)
r2
0.8
0.6
0.4
0.2
rs2373391
ZNF804B→
←C7orf62
88.43
88.44
88.45
88.46
88.47
Position on chr7 (Mb)

date:	Wed Oct 19 17:44:27 2016
build:	hg19
display range:	chr7:88424300−88474300 [88424300−88474300]
hilite range:	0 − 0 [0 − 0]
reference SNP:	chr7:88449300
number of SNPs plotted:	235
min P.value:	4.98E−6 [chr7:88449300]
max P.value:	9.75E−1 [chr7:88425992]

annotation key

framestop	○
splice	□
nonsyn	◇
coding	△
utr	▽
tfbscons	×
mcs44placental	⊠
no annotation	✳
none	⊕

Make more plots at http://csg.sph.umich.edu/locuszoom/

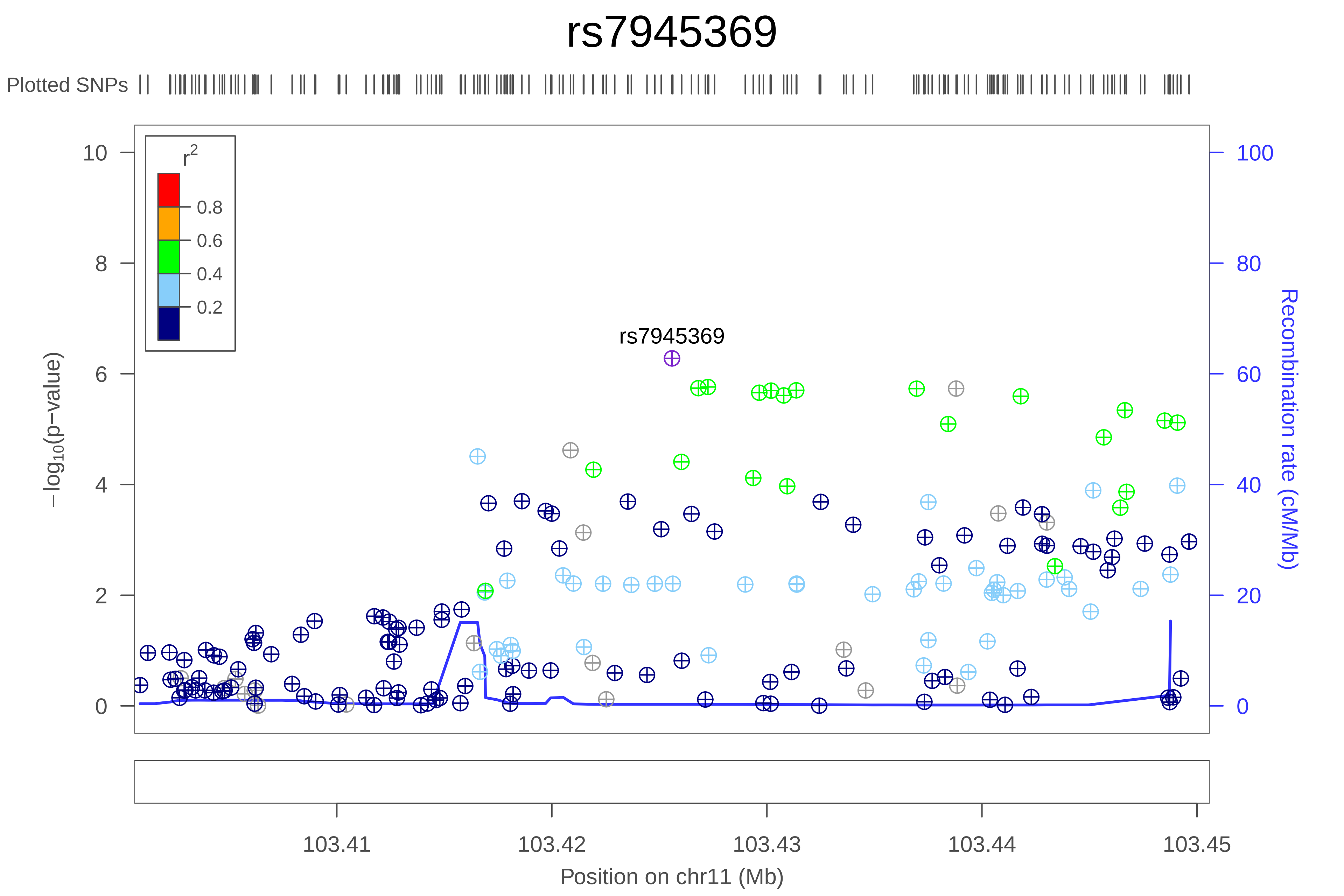

rs7945369
Plotted SNPs
r2
0.8
0.6
0.4
0.2
rs7945369
−log10(p−value)
Recombination rate (cM/Mb)
10
8
6
4
2
0
100
80
60
40
20
0
103.41
103.42
103.43
103.44
103.45
Position on chr11 (Mb)

date: Wed Oct 19 18:02:12 2016

build: hg19

display range: chr11:103400586−103450586 [103400586−103450586]

hilite range: 0 − 0 [0 − 0]

reference SNP: chr11:103425586

number of SNPs plotted: 198

min P.value: 5.26E−7 [chr11:103425586]

max P.value: 9.9E−1 [chr11:103432432]

annotation key

- framestop
- splice
- nonsyn
- coding
- utr
- tfbscons
- mcs44placental
- no annotation
- none

Make more plots at http://csg.sph.umich.edu/locuszoom/
