# Supplementary material for: Genome-wide association study of pigmentary traits (skin and iris color) in individuals of East Asian ancestry
Source: PeerJ. 2017 Nov 2;5:e3951. doi: 10.7717/peerj.3951 (PMC5671666; doi:10.7717/peerj.3951)
Supplement: Figure S8 — All these regions harbour multiple markers showing suggestive significance and good imputation. [file peerj-05-3951-s008.pdf]

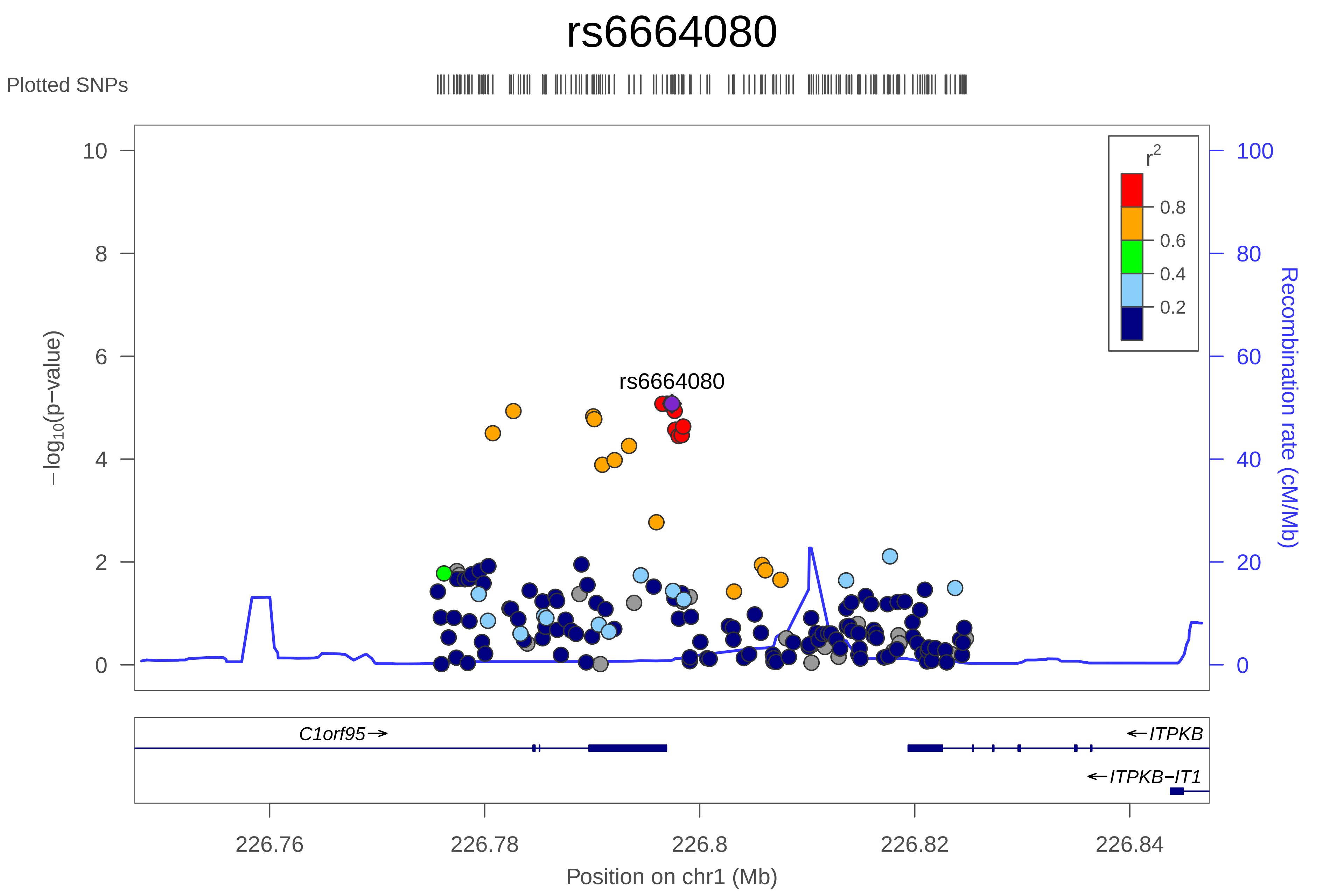
rs6664080
Plotted SNPs
10
8
6
4
2
0
−log10(p−value)
100
80
60
40
20
0
Recombination rate (cM/Mb)
r2
0.8
0.6
0.4
0.2
rs6664080
C1orf95→
←ITPKB
←ITPKB−IT1
226.76
226.78
226.8
226.82
226.84
Position on chr1 (Mb)

date:	Thu Jul 27 23:57:52 2017
build:	hg19
display range:	chr1:226747426−226847426 [226747426−226847426]
hilite range:	0 − 0 [0 − 0]
reference SNP:	chr1:226797426
number of SNPs plotted:	166
min P.value:	8.36E−6 [chr1:226796968]
max P.value:	9.66E−1 [chr1:226790769]

Make more plots at http://csg.sph.umich.edu/locuszoom/

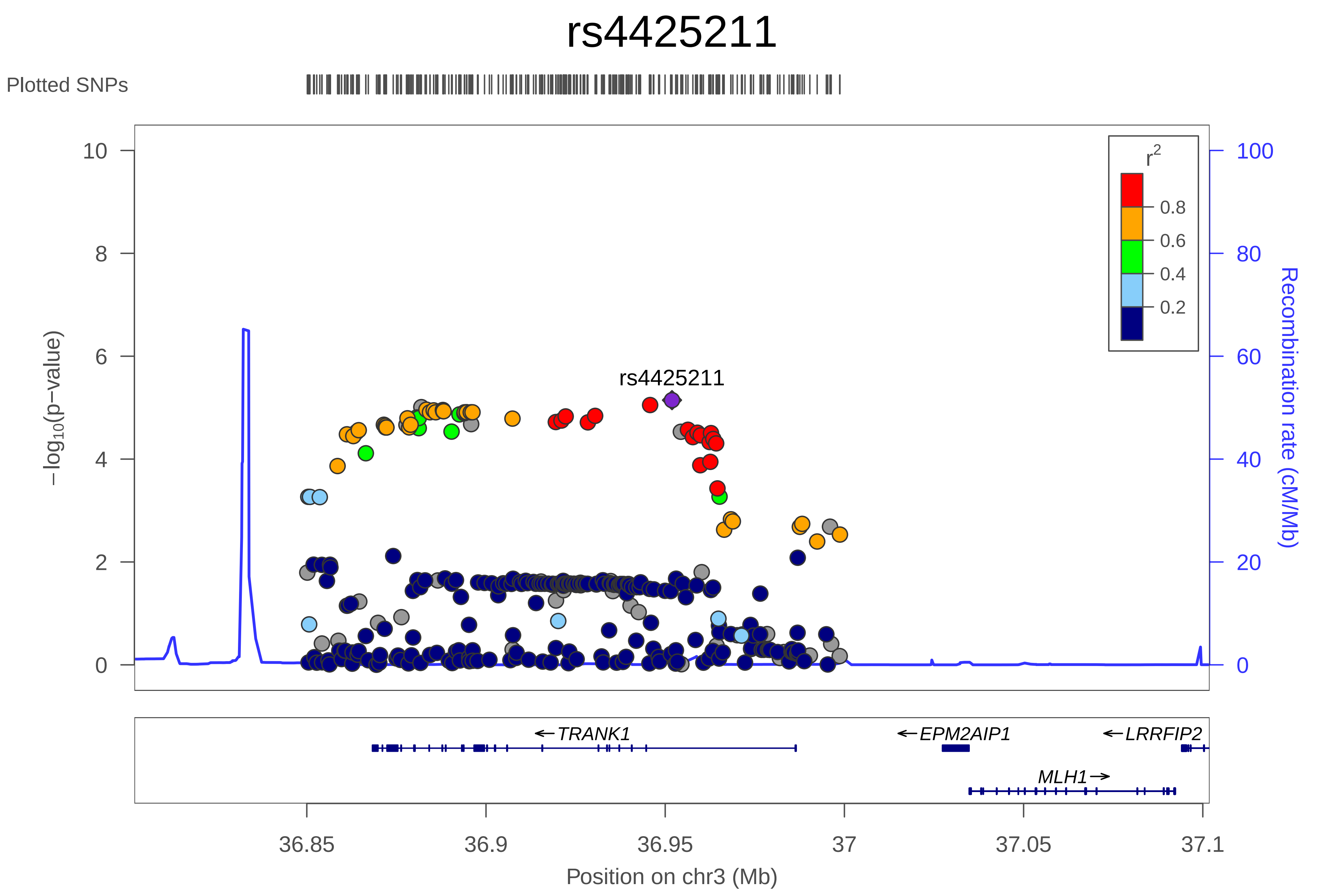

rs4425211
Plotted SNPs
10
8
6
4
2
0
−log10(p−value)
100
80
60
40
20
0
Recombination rate (cM/Mb)
r2
0.8
0.6
0.4
0.2
rs4425211
←TRANK1
←EPM2AIP1
←LRRFIP2
MLH1→
36.85
36.9
36.95
37
37.05
37.1
Position on chr3 (Mb)

date:	Thu Jul 27 23:48:08 2017
build:	hg19
display range:	chr3:36801898−37101898 [36801898−37101898]
hilite range:	0 − 0 [0 − 0]
reference SNP:	chr3:36951898
number of SNPs plotted:	304
min P.value:	7.14E−6 [chr3:36951898]
max P.value:	9.95E−1 [chr3:36869439]

Make more plots at http://csg.sph.umich.edu/locuszoom/

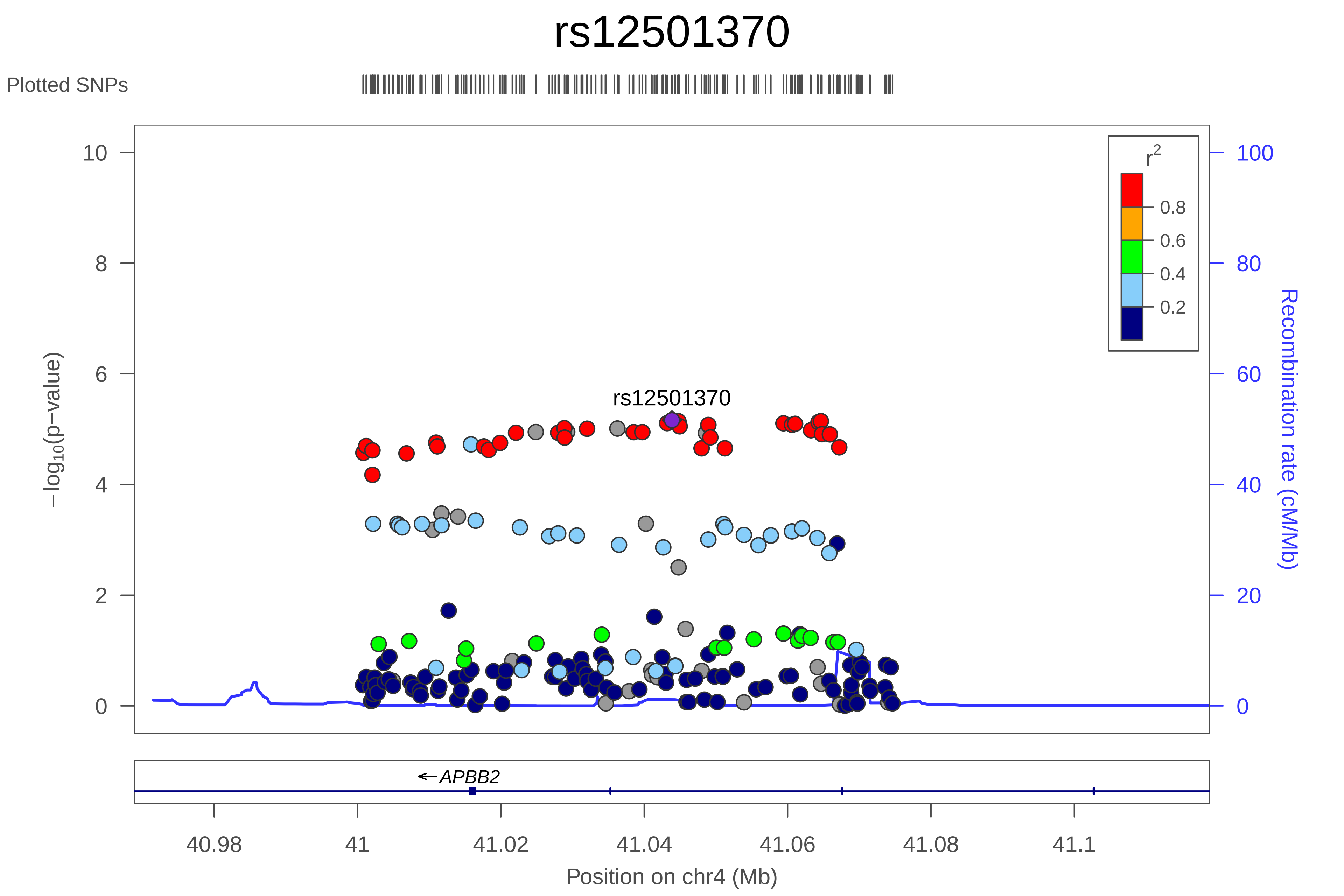

rs12501370
Plotted SNPs
10
8
6
4
2
0
−log10(p−value)
100
80
60
40
20
0
Recombination rate (cM/Mb)
r2
0.8
0.6
0.4
0.2
rs12501370
←APBB2
40.98
41
41.02
41.04
41.06
41.08
41.1
Position on chr4 (Mb)

date:	Fri Jul 28 00:19:10 2017
build:	hg19
display range:	chr4:40968870−41118870 [40968870−41118870]
hilite range:	0 − 0 [0 − 0]
reference SNP:	chr4:41043870
number of SNPs plotted:	204
min P.value:	6.89E−6 [chr4:41043870]
max P.value:	9.91E−1 [chr4:41067995]

Make more plots at http://csg.sph.umich.edu/locuszoom/

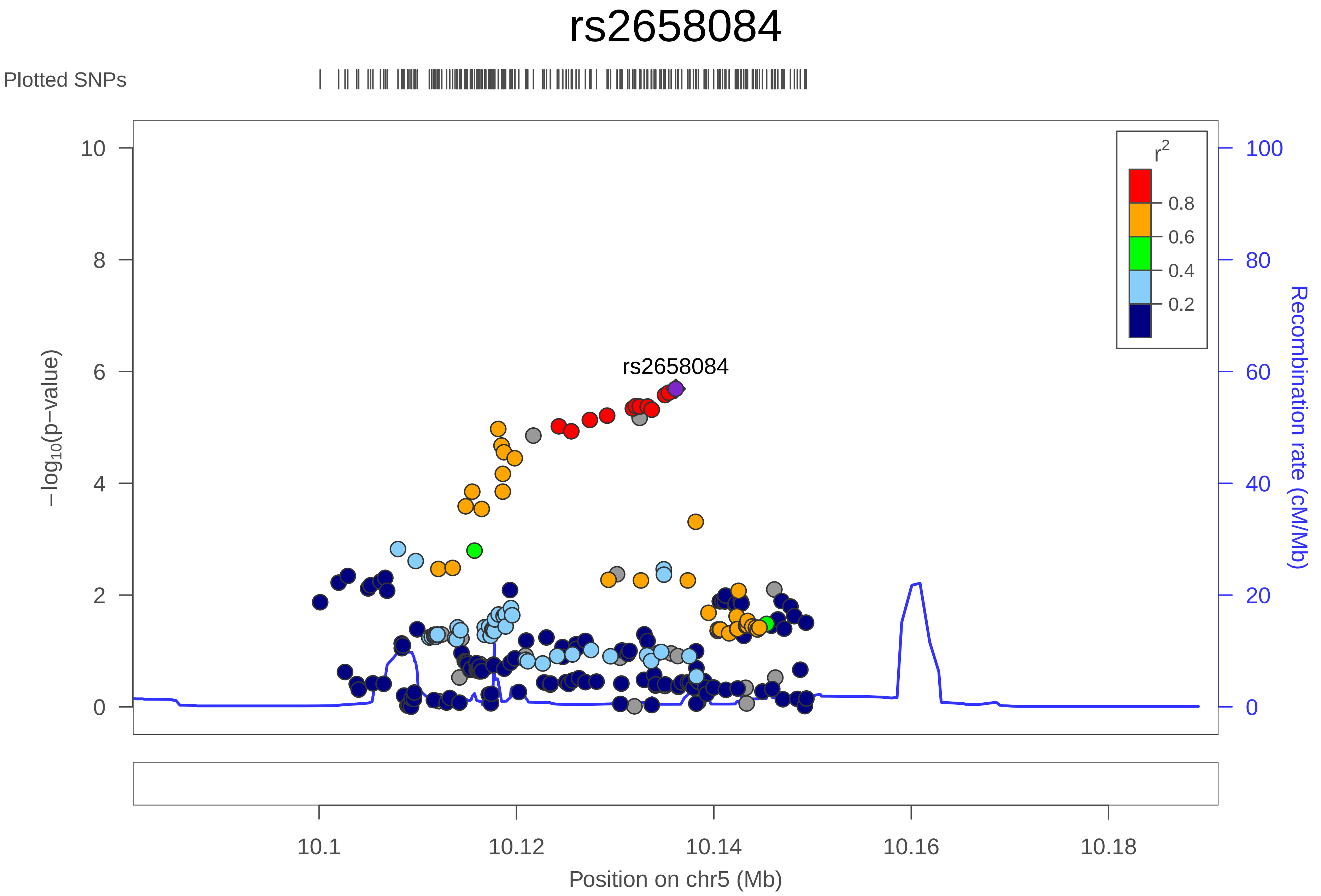
rs2658084
Plotted SNPs
10
8
6
4
2
0
−log10(p−value)
100
80
60
40
20
0
Recombination rate (cM/Mb)
r2
0.8
0.6
0.4
0.2
rs2658084
10.1
10.12
10.14
10.16
10.18
Position on chr5 (Mb)

date:	Thu Jul 27 23:37:10 2017
build:	hg19
display range:	chr5:10081135−10191135 [10081135−10191135]
hilite range:	0 − 0 [0 − 0]
reference SNP:	chr5:10136135
number of SNPs plotted:	238
min P.value:	2.05E−6 [chr5:10136135]
max P.value:	9.92E−1 [chr5:10109323]

Make more plots at http://csg.sph.umich.edu/locuszoom/

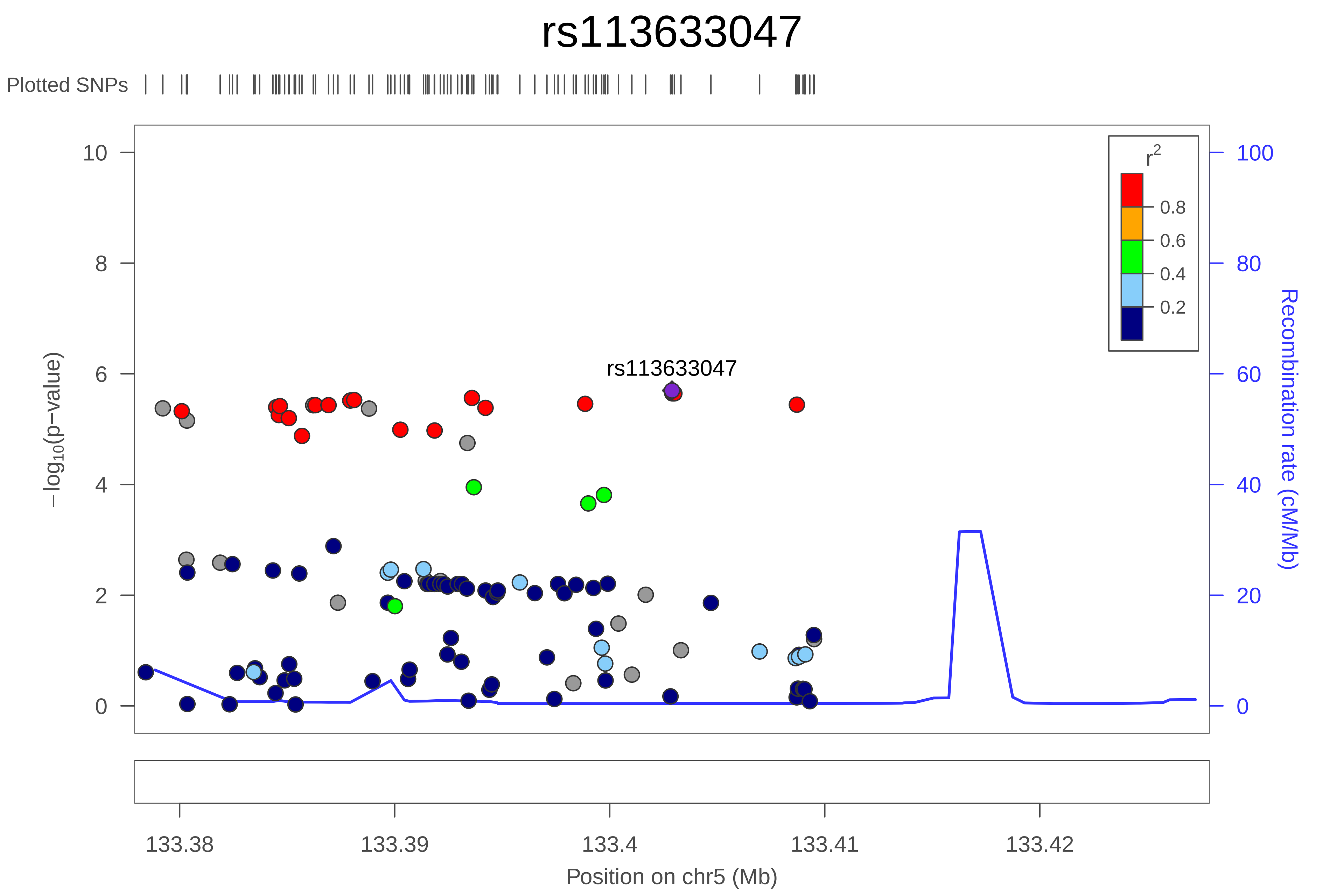
rs113633047
Plotted SNPs
rs113633047
r^2
0.8
0.6
0.4
0.2
10
8
6
4
2
0
−log10(p−value)
100
80
60
40
20
0
Recombination rate (cM/Mb)
133.38
133.39
133.4
133.41
133.42
Position on chr5 (Mb)

date:	Mon Aug 7 13:42:39 2017
build:	hg19
display range:	chr5:133377895−133427895 [133377895−133427895]
hilite range:	0 − 0 [0 − 0]
reference SNP:	chr5:133402895
number of SNPs plotted:	110
min p−value:	2E−6 [chr5:133402895]
max p−value:	9.41E−1 [chr5:133385389]

Make more plots at http://csg.sph.umich.edu/locuszoom/

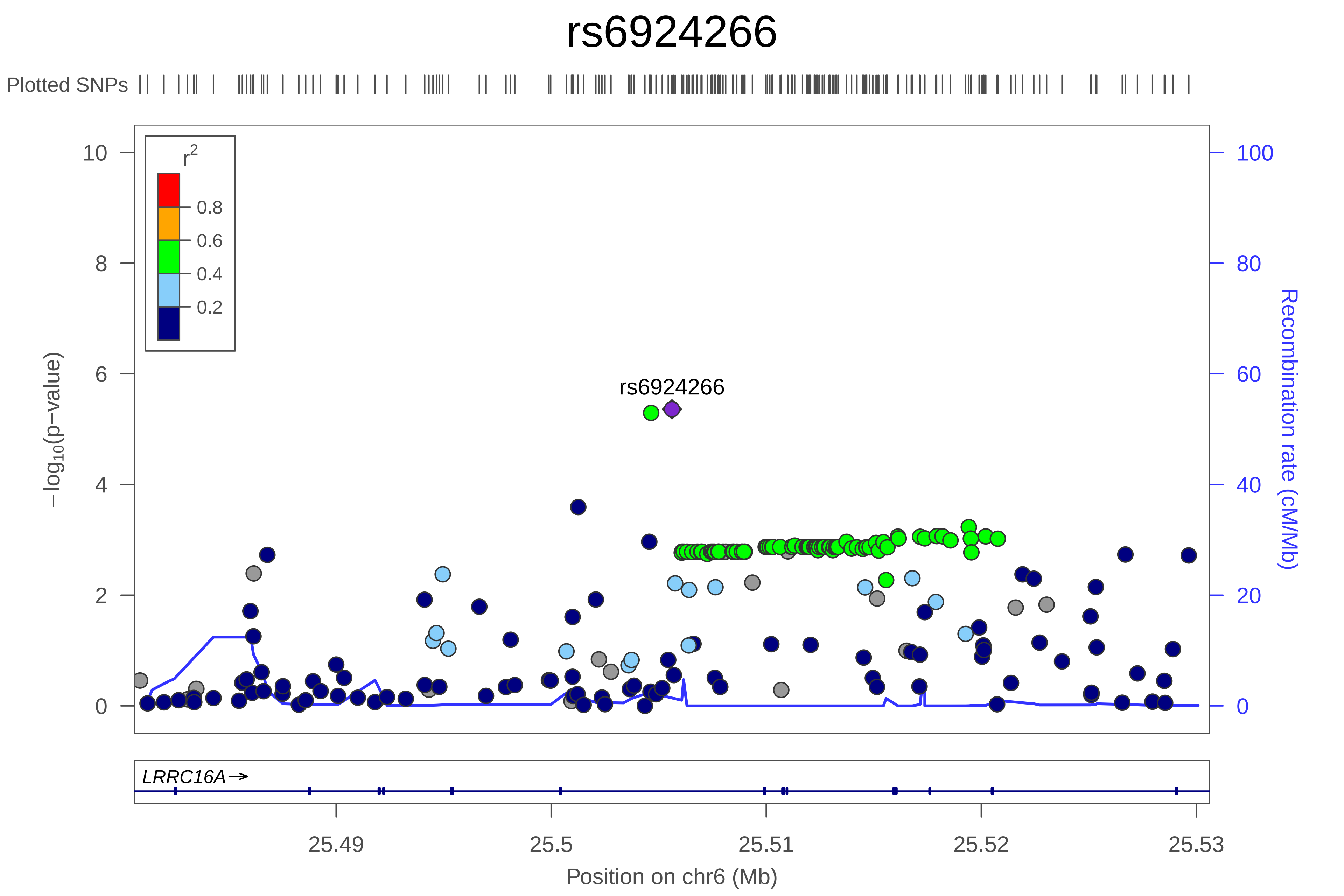
rs6924266
Plotted SNPs
r2
0.8
0.6
0.4
0.2
rs6924266
−log10(p−value)
Recombination rate (cM/Mb)
0
2
4
6
8
10
0
20
40
60
80
100
LRRC16A→
25.49
25.5
25.51
25.52
25.53
Position on chr6 (Mb)

date:	Mon Aug 7 13:45:33 2017
build:	hg19
display range:	chr6:25480617−25530617 [25480617−25530617]
hilite range:	0 − 0 [0 − 0]
reference SNP:	chr6:25505617
number of SNPs plotted:	207
min p−value:	4.38E−6 [chr6:25505617]
max p−value:	9.99E−1 [chr6:25504357]

Make more plots at http://csg.sph.umich.edu/locuszoom/

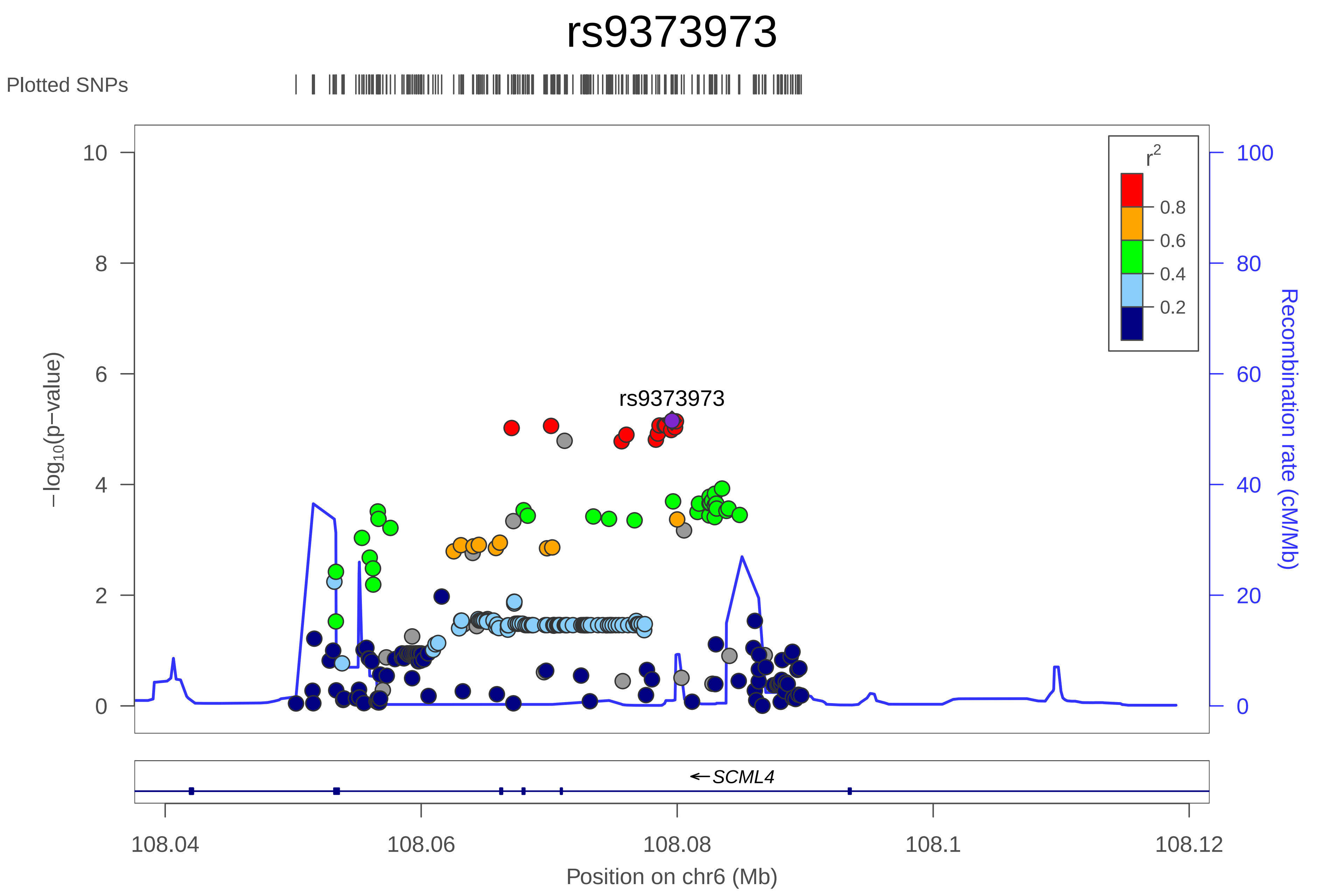
rs9373973
Plotted SNPs
−log10(p−value)
Recombination rate (cM/Mb)
r2
0.8
0.6
0.4
0.2
rs9373973
SCML4
108.04
108.06
108.08
108.1
108.12
Position on chr6 (Mb)
0
2
4
6
8
10
0
20
40
60
80
100

date:	Fri Jul 28 00:10:42 2017
build:	hg19
display range:	chr6:108037595−108121595 [108037595−108121595]
hilite range:	0 − 0 [0 − 0]
reference SNP:	chr6:108079595
number of SNPs plotted:	252
min P.value:	7.02E−6 [chr6:108079595]
max P.value:	9.93E−1 [chr6:108086659]

Make more plots at http://csg.sph.umich.edu/locuszoom/

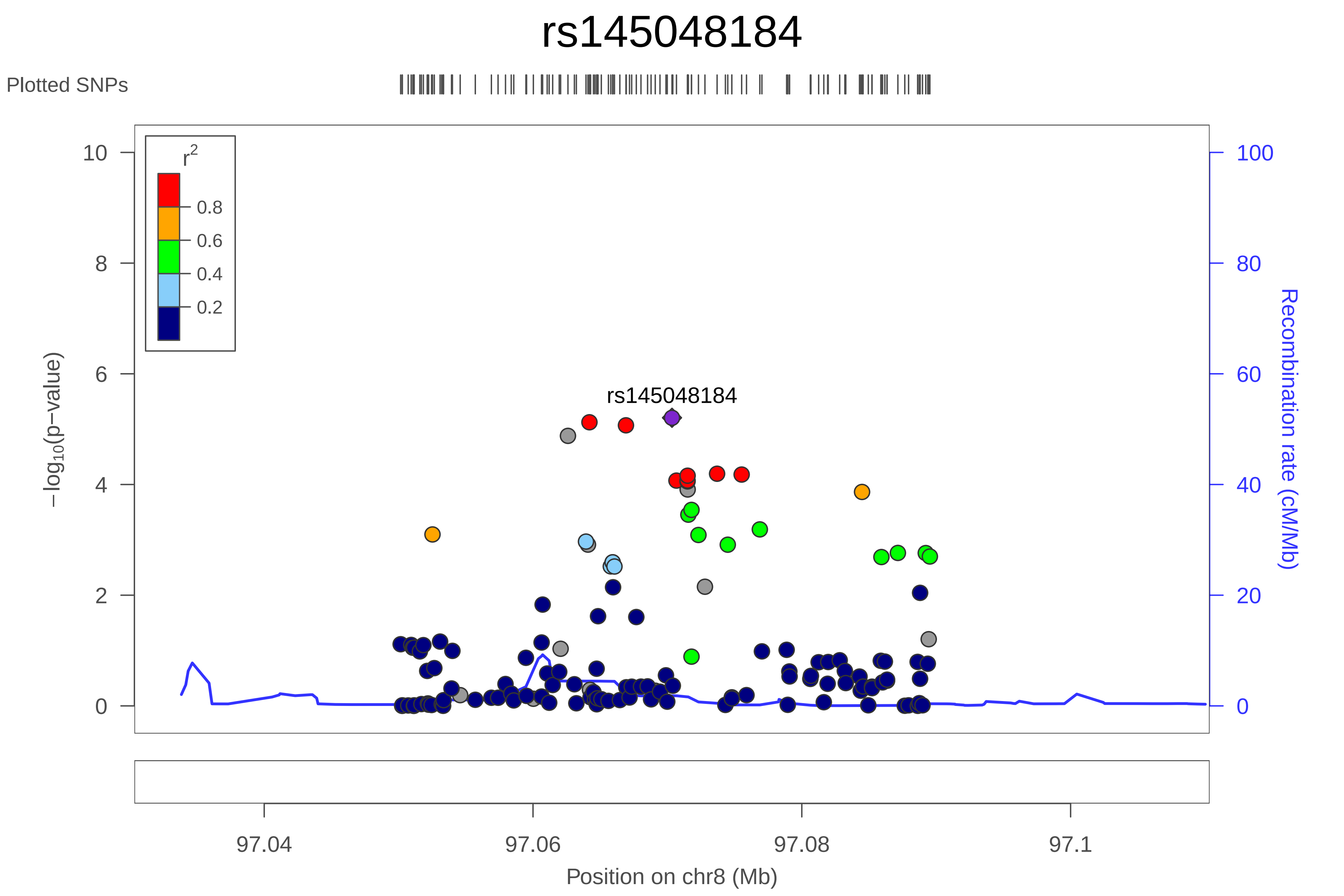
rs145048184
Plotted SNPs
r2
0.8
0.6
0.4
0.2
rs145048184
−log10(p−value)
10
8
6
4
2
0
100
80
60
40
20
0
Recombination rate (cM/Mb)
97.04
97.06
97.08
97.1
Position on chr8 (Mb)

date:	Fri Jul 28 00:44:54 2017
build:	hg19
display range:	chr8:97030341−97110341 [97030341−97110341]
hilite range:	0 − 0 [0 − 0]
reference SNP:	chr8:97070341
number of SNPs plotted:	137
min P.value:	6.23E−6 [chr8:97070341]
max P.value:	9.99E−1 [chr8:97051128]

Make more plots at http://csg.sph.umich.edu/locuszoom/

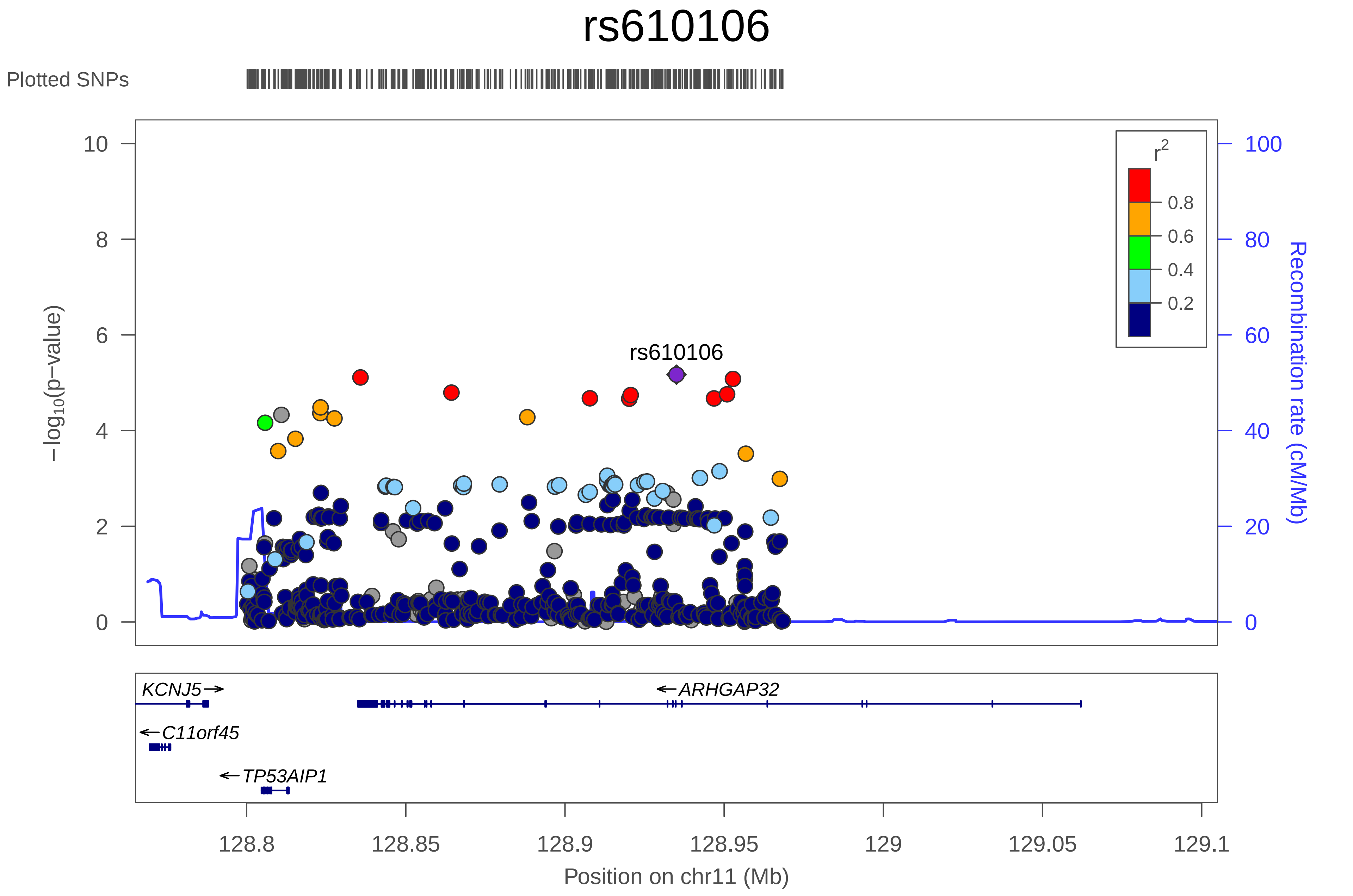

rs610106
Plotted SNPs
−log10(p−value)
Recombination rate (cM/Mb)
r2
0.8
0.6
0.4
0.2
rs610106
KCNJ5
ARHGAP32
C11orf45
TP53AIP1
128.8
128.85
128.9
128.95
129
129.05
129.1
Position on chr11 (Mb)

date:	Wed Jul 26 05:06:53 2017
build:	hg19
display range:	chr11:128765028−129105028 [128765028−129105028]
hilite range:	0 − 0 [0 − 0]
reference SNP:	chr11:128935028
number of SNPs plotted:	435
min P.value:	6.79E−6 [chr11:128935028]
max P.value:	9.88E−1 [chr11:128912952]

Make more plots at http://csg.sph.umich.edu/locuszoom/

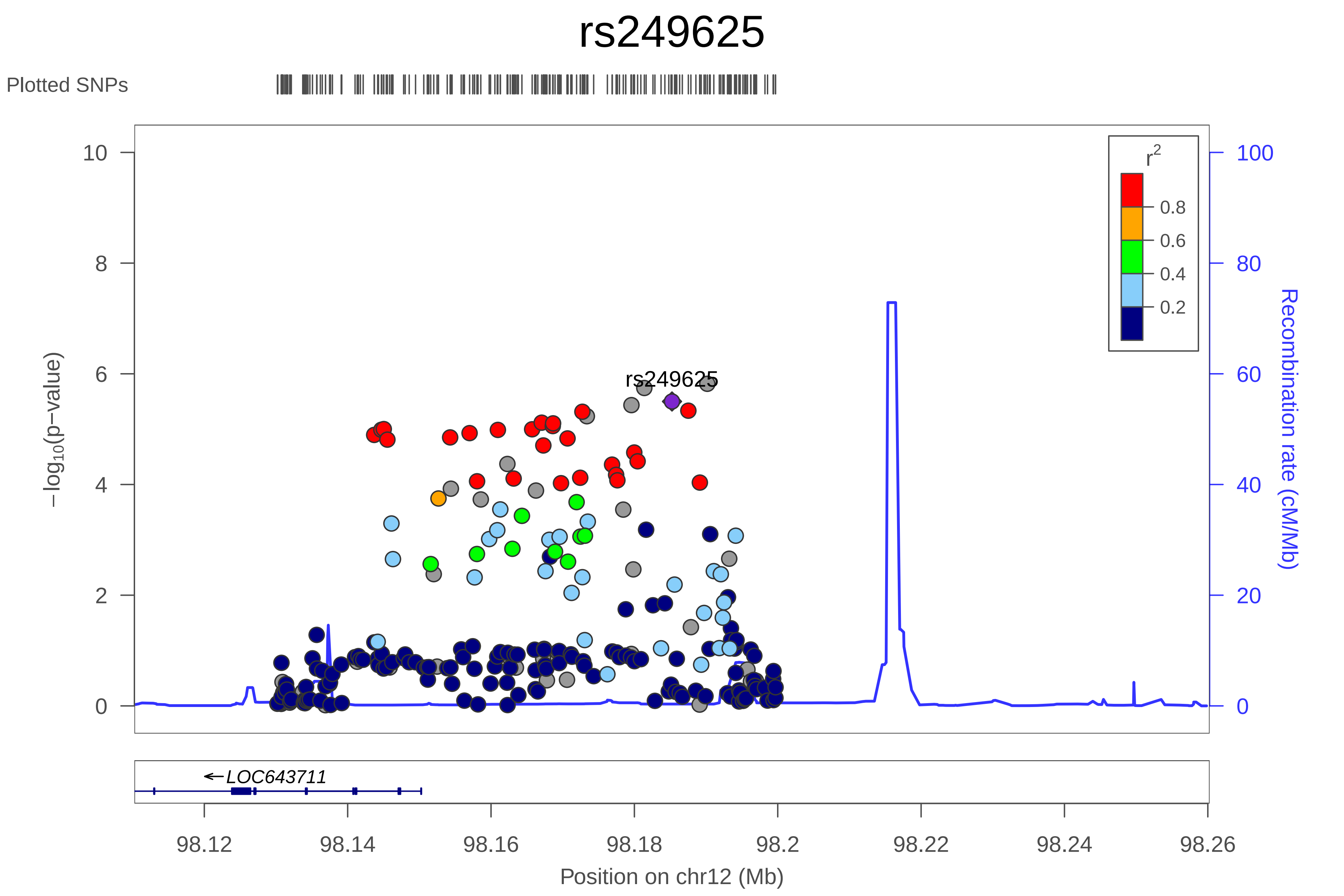
rs249625
Plotted SNPs
−log10(p−value)
Recombination rate (cM/Mb)
r2
0.8
0.6
0.4
0.2
rs249625
10
8
6
4
2
0
100
80
60
40
20
0
LOC643711
98.12
98.14
98.16
98.18
98.2
98.22
98.24
98.26
Position on chr12 (Mb)

date:	Thu Jul 27 23:28:54 2017
build:	hg19
display range:	chr12:98110248−98260248 [98110248−98260248]
hilite range:	0 − 0 [0 − 0]
reference SNP:	chr12:98185248
number of SNPs plotted:	231
min P.value:	1.51E−6 [chr12:98190174]
max P.value:	9.7E−1 [chr12:98136905]

Make more plots at http://csg.sph.umich.edu/locuszoom/

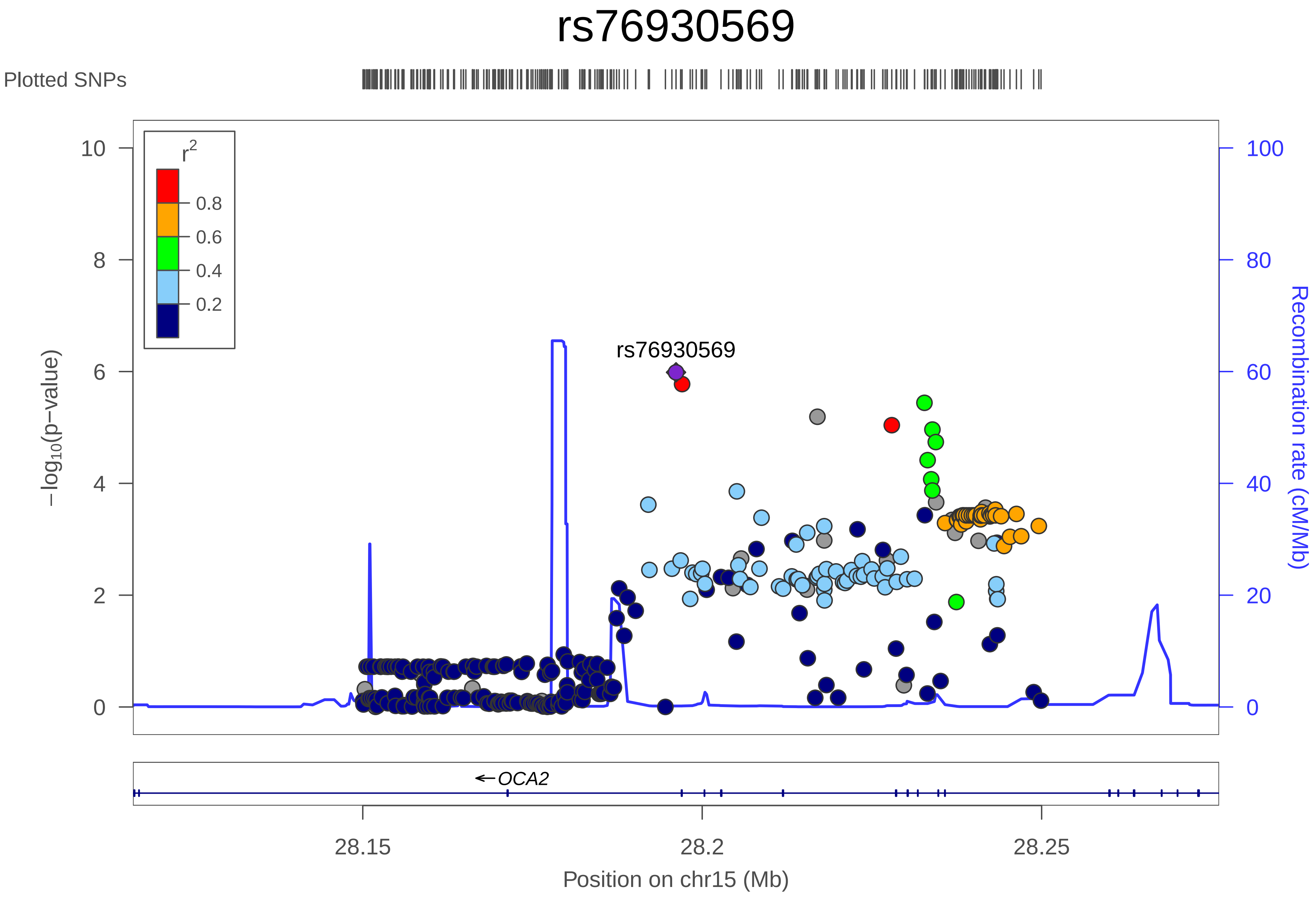
rs76930569
Plotted SNPs
r2
0.8
0.6
0.4
0.2
rs76930569
10
8
6
4
2
0
−log10(p−value)
100
80
60
40
20
0
Recombination rate (cM/Mb)
← OCA2
28.15
28.2
28.25
Position on chr15 (Mb)

date:	Fri Jul 28 00:34:11 2017
build:	hg19
display range:	chr15:28116145−28276145 [28116145−28276145]
hilite range:	0 − 0 [0 − 0]
reference SNP:	chr15:28196145
number of SNPs plotted:	303
min P.value:	1.03E−6 [chr15:28196145]
max P.value:	9.97E−1 [chr15:28194580]

Make more plots at http://csg.sph.umich.edu/locuszoom/

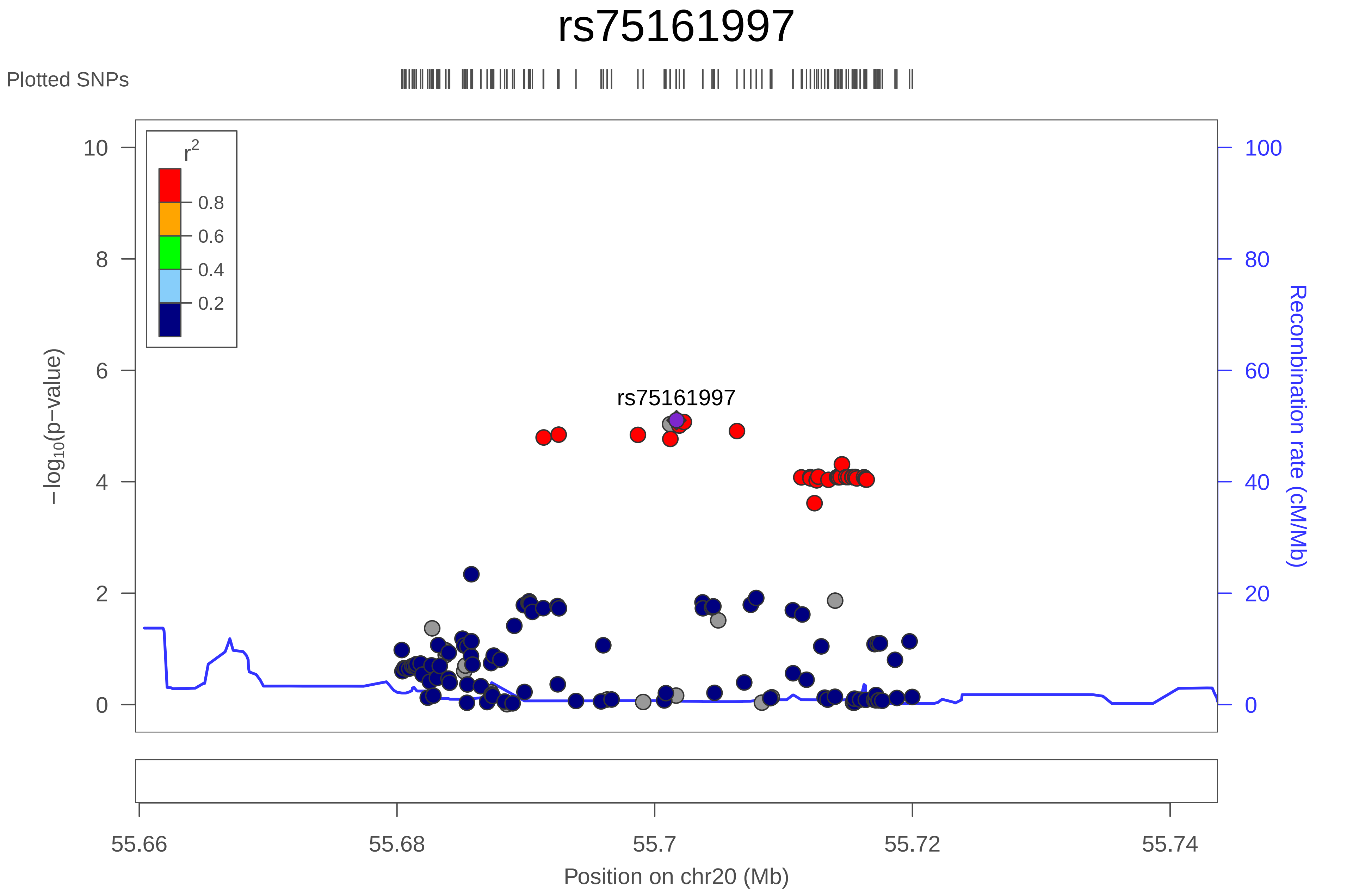

rs75161997
Plotted SNPs
r2
0.8
0.6
0.4
0.2
10
8
6
4
2
0
−log10(p−value)
100
80
60
40
20
0
Recombination rate (cM/Mb)
rs75161997
55.66
55.68
55.7
55.72
55.74
Position on chr20 (Mb)

date:	Fri Jul 28 00:24:33 2017
build:	hg19
display range:	chr20:55659691−55743691 [55659691−55743691]
hilite range:	0 − 0 [0 − 0]
reference SNP:	chr20:55701691
number of SNPs plotted:	144
min P.value:	7.84E−6 [chr20:55701691]
max P.value:	9.82E−1 [chr20:55688533]

Make more plots at http://csg.sph.umich.edu/locuszoom/

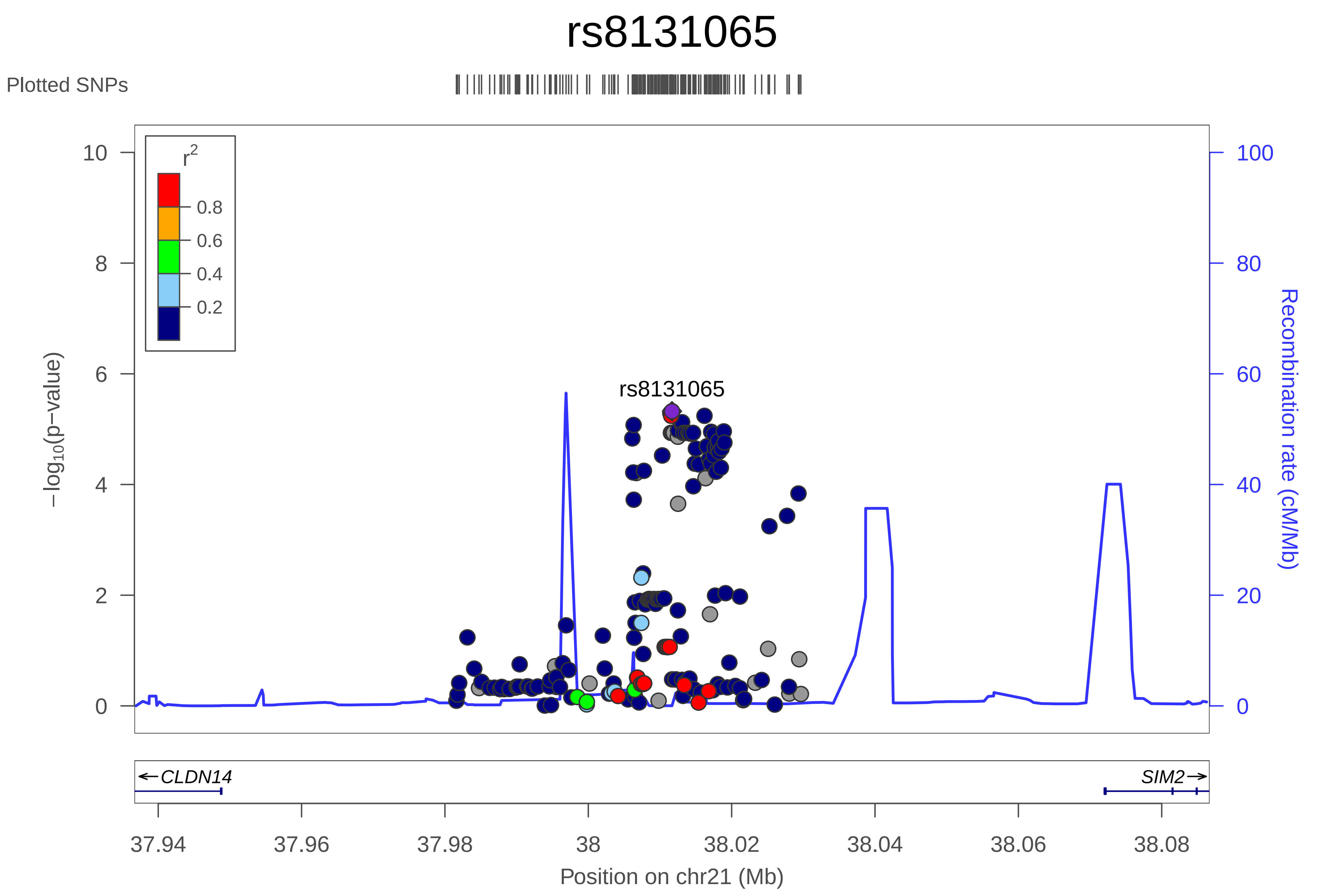
rs8131065
Plotted SNPs
r2
0.8
0.6
0.4
0.2
10
8
6
4
2
0
−log10(p−value)
100
80
60
40
20
0
Recombination rate (cM/Mb)
rs8131065
←CLDN14
SIM2→
37.94
37.96
37.98
38
38.02
38.04
38.06
38.08
Position on chr21 (Mb)

date:	Fri Jul 28 00:05:43 2017
build:	hg19
display range:	chr21:37936676−38086676 [37936676−38086676]
hilite range:	0 − 0 [0 − 0]
reference SNP:	chr21:38011676
number of SNPs plotted:	180
min P.value:	4.75E−6 [chr21:38011676]
max P.value:	9.86E−1 [chr21:37993934]

Make more plots at http://csg.sph.umich.edu/locuszoom/
