# Supplementary material for: Genome-wide association study of pigmentary traits (skin and iris color) in individuals of East Asian ancestry
Source: PeerJ. 2017 Nov 2;5:e3951. doi: 10.7717/peerj.3951 (PMC5671666; doi:10.7717/peerj.3951)
Supplement: Figure S9 — All these regions harbour multiple markers showing suggestive significance and good imputation scores (e.g., score info > 0.8). [file peerj-05-3951-s009.pdf]

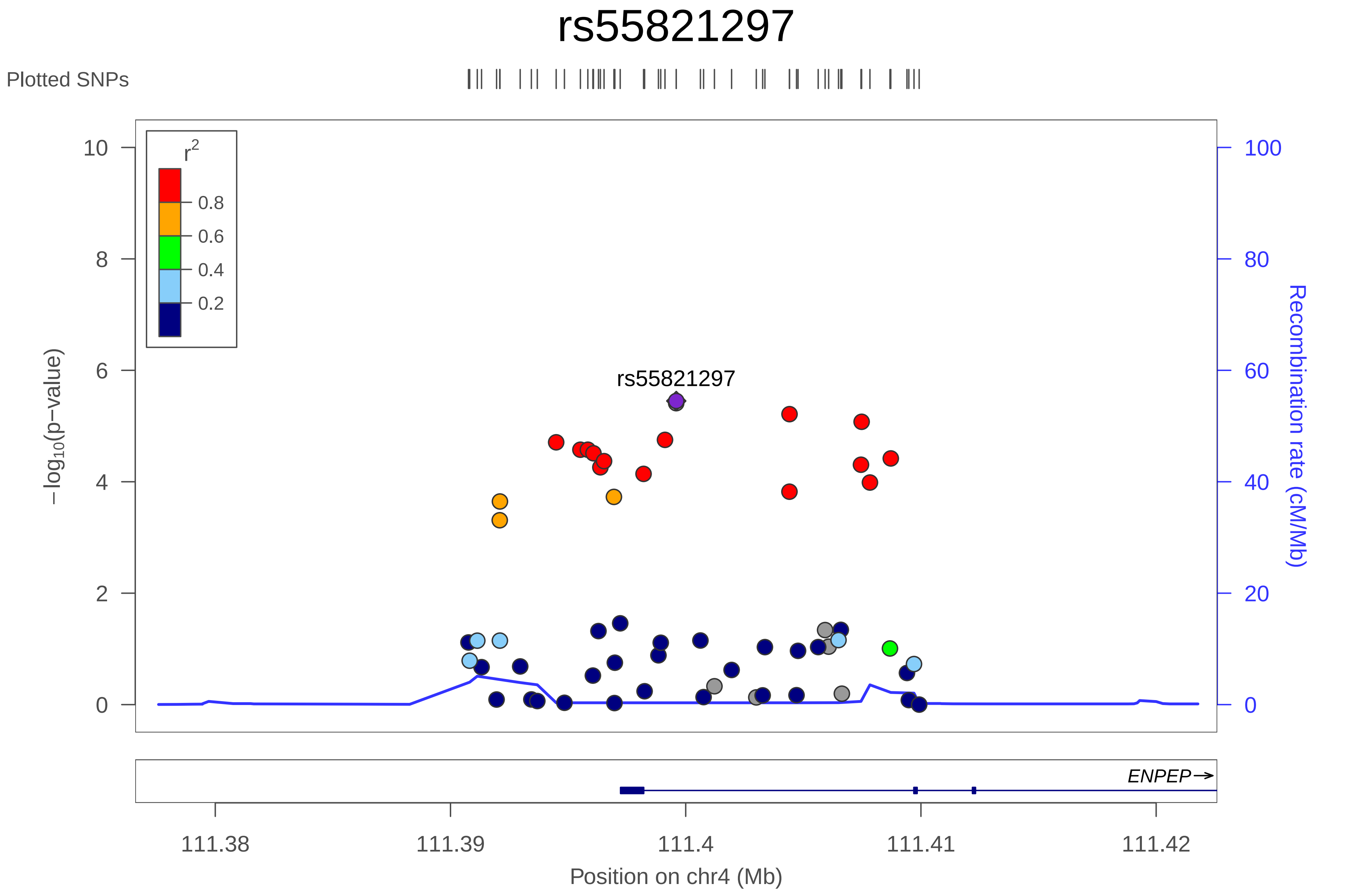

rs55821297
Plotted SNPs
r2
0.8
0.6
0.4
0.2
rs55821297
−log10(p−value)
10
8
6
4
2
0
100
80
60
40
20
0
Recombination rate (cM/Mb)
ENPEP→
111.38
111.39
111.4
111.41
111.42
Position on chr4 (Mb)

date:	Fri Jul 28 01:13:34 2017
build:	hg19
display range:	chr4:111376598−111422598 [111376598−111422598]
hilite range:	0 − 0 [0 − 0]
reference SNP:	chr4:111399598
number of SNPs plotted:	58
min P.value:	3.55E−6 [chr4:111399598]
max P.value:	9.98E−1 [chr4:111409925]

Make more plots at http://csg.sph.umich.edu/locuszoom/

rs72763726

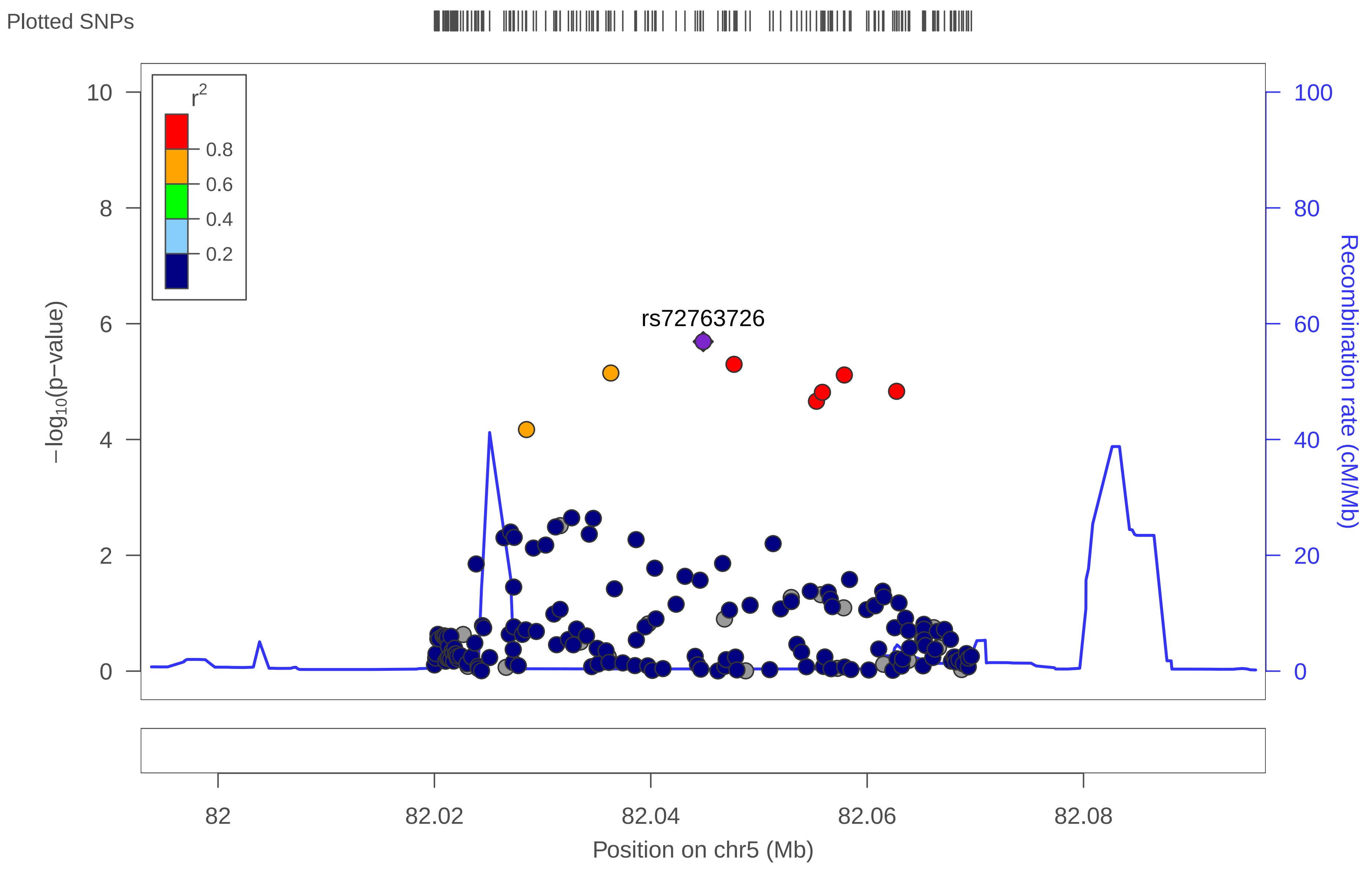
Plotted SNPs
r2
0.8
0.6
0.4
0.2
rs72763726
−log10(p−value)
Recombination rate (cM/Mb)
10
8
6
4
2
0
100
80
60
40
20
0
82
82.02
82.04
82.06
82.08
Position on chr5 (Mb)

date:	Fri Jul 28 01:56:56 2017
build:	hg19
display range:	chr5:81992846−82096846 [81992846−82096846]
hilite range:	0 − 0 [0 − 0]
reference SNP:	chr5:82044846
number of SNPs plotted:	175
min P.value:	2.04E−6 [chr5:82044846]
max P.value:	9.92E−1 [chr5:82046207]

Make more plots at http://csg.sph.umich.edu/locuszoom/

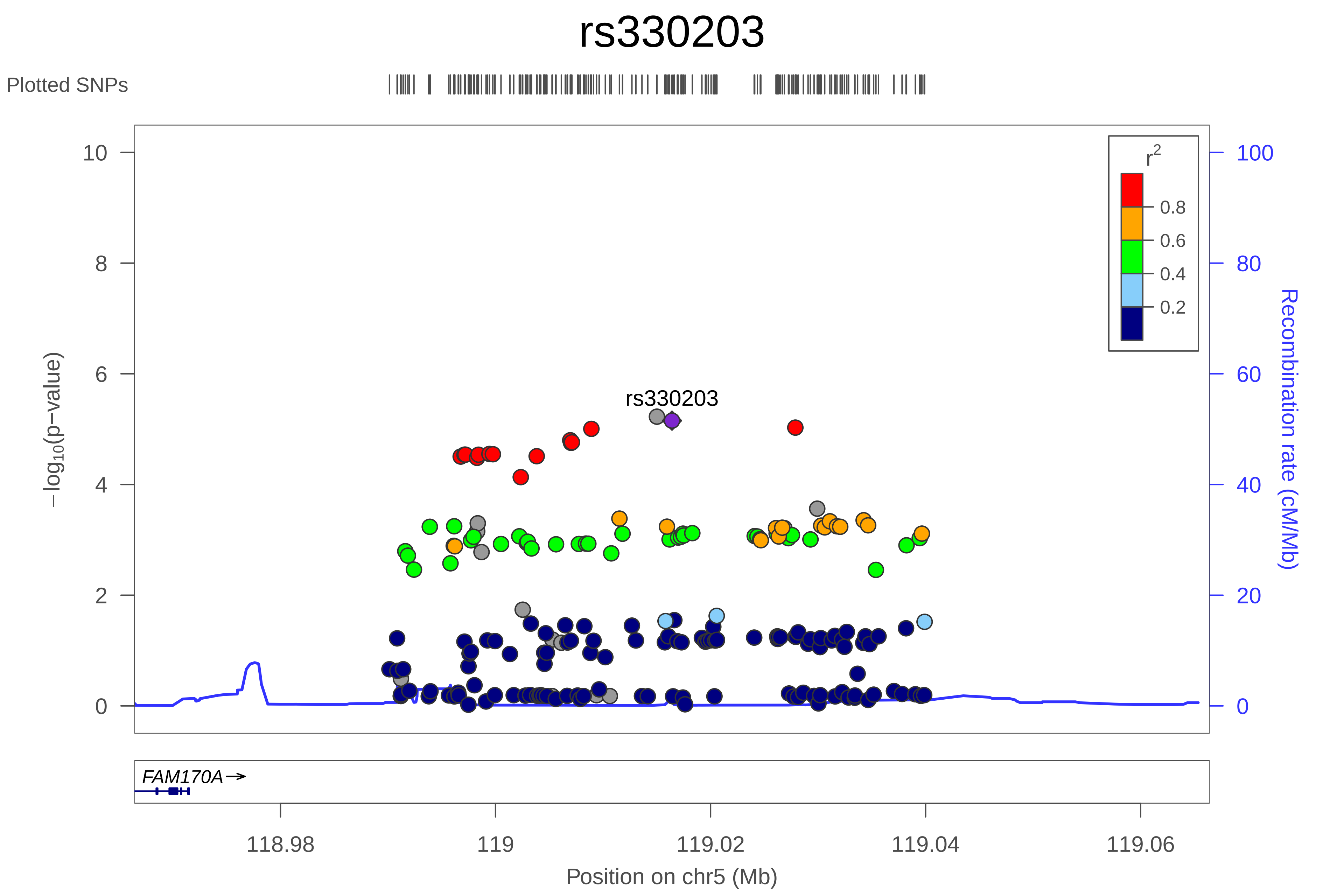
rs330203
Plotted SNPs
r2
0.8
0.6
0.4
0.2
rs330203
−log10(p−value)
Recombination rate (cM/Mb)
10
8
6
4
2
0
100
80
60
40
20
0
FAM170A→
118.98
119
119.02
119.04
119.06
Position on chr5 (Mb)

date:	Fri Jul 28 01:03:38 2017
build:	hg19
display range:	chr5:118966414−119066414 [118966414−119066414]
hilite range:	0 − 0 [0 − 0]
reference SNP:	chr5:119016414
number of SNPs plotted:	198
min P.value:	5.94E−6 [chr5:119015012]
max P.value:	9.51E−1 [chr5:118997478]

Make more plots at http://csg.sph.umich.edu/locuszoom/

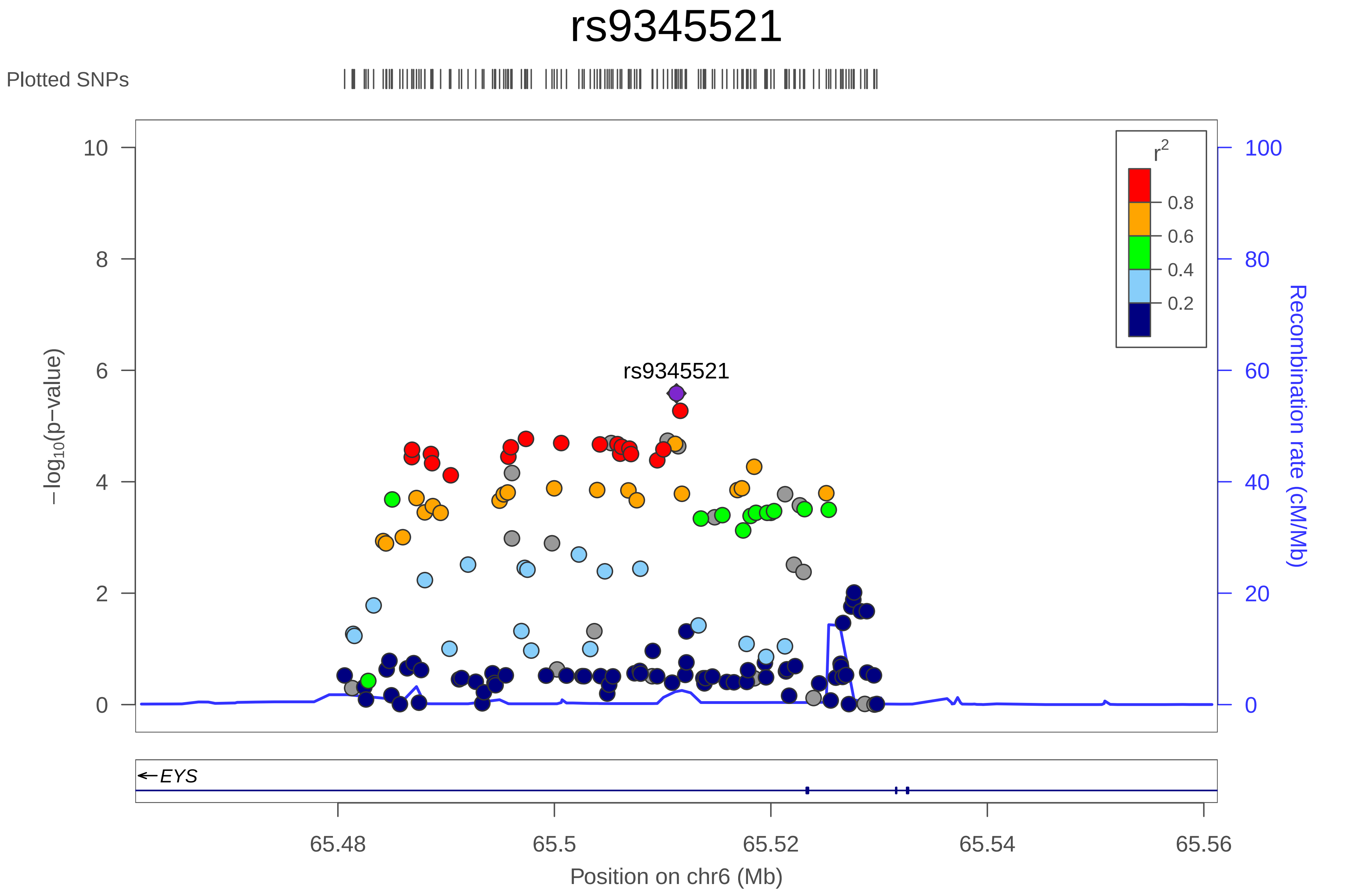
rs9345521
Plotted SNPs
rs9345521
−log10(p−value)
Recombination rate (cM/Mb)
r2
0.8
0.6
0.4
0.2
EYS
65.48
65.5
65.52
65.54
65.56
Position on chr6 (Mb)
0
2
4
6
8
10
0
20
40
60
80
100

date:	Fri Jul 28 02:12:33 2017
build:	hg19
display range:	chr6:65461281−65561281 [65461281−65561281]
hilite range:	0 − 0 [0 − 0]
reference SNP:	chr6:65511281
number of SNPs plotted:	157
min P.value:	2.59E−6 [chr6:65511281]
max P.value:	9.97E−1 [chr6:65529577]

Make more plots at http://csg.sph.umich.edu/locuszoom/

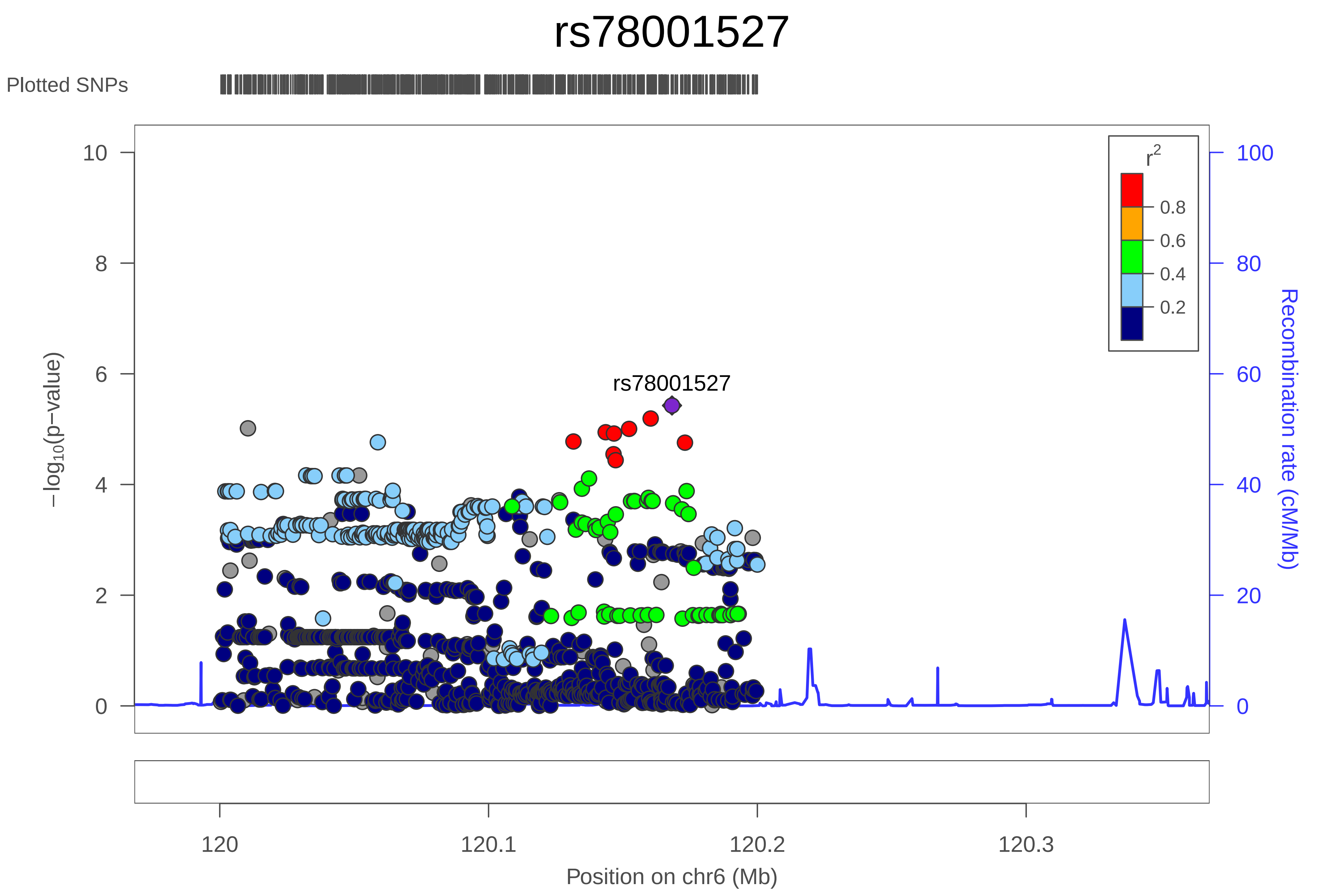

rs78001527
Plotted SNPs
10
8
6
4
2
0
−log10(p−value)
100
80
60
40
20
0
Recombination rate (cM/Mb)
r2
0.8
0.6
0.4
0.2
rs78001527
120
120.1
120.2
120.3
Position on chr6 (Mb)

date:	Fri Jul 28 02:07:52 2017
build:	hg19
display range:	chr6:119968240−120368240 [119968240−120368240]
hilite range:	0 − 0 [0 − 0]
reference SNP:	chr6:120168240
number of SNPs plotted:	929
min P.value:	3.74E−6 [chr6:120168240]
max P.value:	9.98E−1 [chr6:120103856]

Make more plots at http://csg.sph.umich.edu/locuszoom/

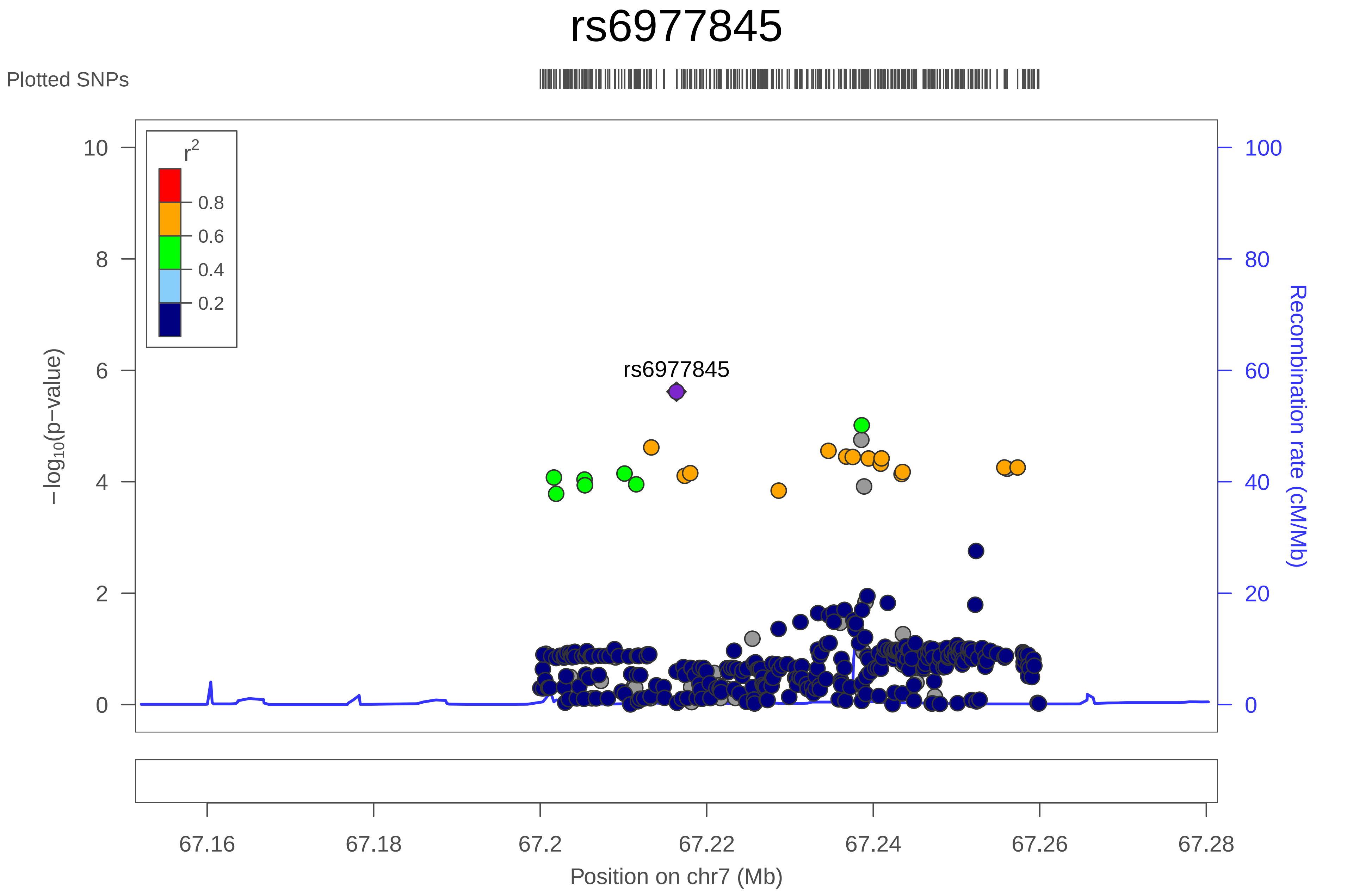
rs6977845
Plotted SNPs
r2
0.8
0.6
0.4
0.2
rs6977845
−log10(p−value)
Recombination rate (cM/Mb)
10
8
6
4
2
0
100
80
60
40
20
0
67.16
67.18
67.2
67.22
67.24
67.26
67.28
Position on chr7 (Mb)

date:	Fri Jul 28 01:50:05 2017
build:	hg19
display range:	chr7:67151370−67281370 [67151370−67281370]
hilite range:	0 − 0 [0 − 0]
reference SNP:	chr7:67216370
number of SNPs plotted:	355
min P.value:	2.42E−6 [chr7:67216370]
max P.value:	9.89E−1 [chr7:67210827]

Make more plots at http://csg.sph.umich.edu/locuszoom/

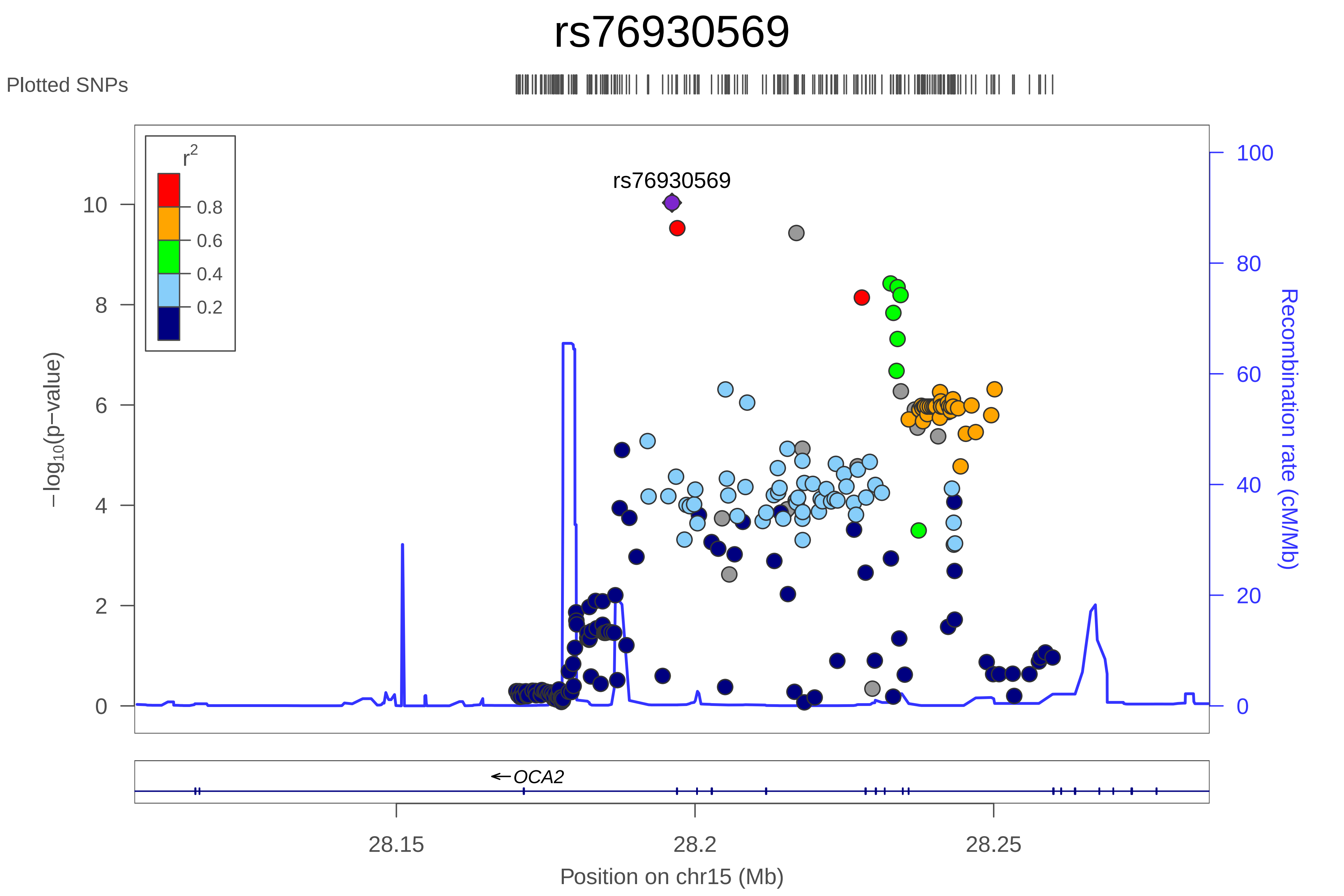
rs76930569
Plotted SNPs
r2
0.8
0.6
0.4
0.2
rs76930569
10
8
6
4
2
0
−log10(p−value)
100
80
60
40
20
0
Recombination rate (cM/Mb)
← OCA2
28.15
28.2
28.25
Position on chr15 (Mb)

date:	Fri Jul 28 02:01:21 2017
build:	hg19
display range:	chr15:28106145−28286145 [28106145−28286145]
hilite range:	0 − 0 [0 − 0]
reference SNP:	chr15:28196145
number of SNPs plotted:	221
min P.value:	9.27E−11 [chr15:28196145]
max P.value:	8.49E−1 [chr15:28218295]

Make more plots at http://csg.sph.umich.edu/locuszoom/
