# Supplementary material for: Genome-wide association study of pigmentary traits (skin and iris color) in individuals of East Asian ancestry
Source: PeerJ. 2017 Nov 2;5:e3951. doi: 10.7717/peerj.3951 (PMC5671666; doi:10.7717/peerj.3951)
Supplement: Figure S10 — All these regions harbour multiple markers showing suggestive significance and good imputation scores (e.g., score info > 0.8). [file peerj-05-3951-s010.pdf]

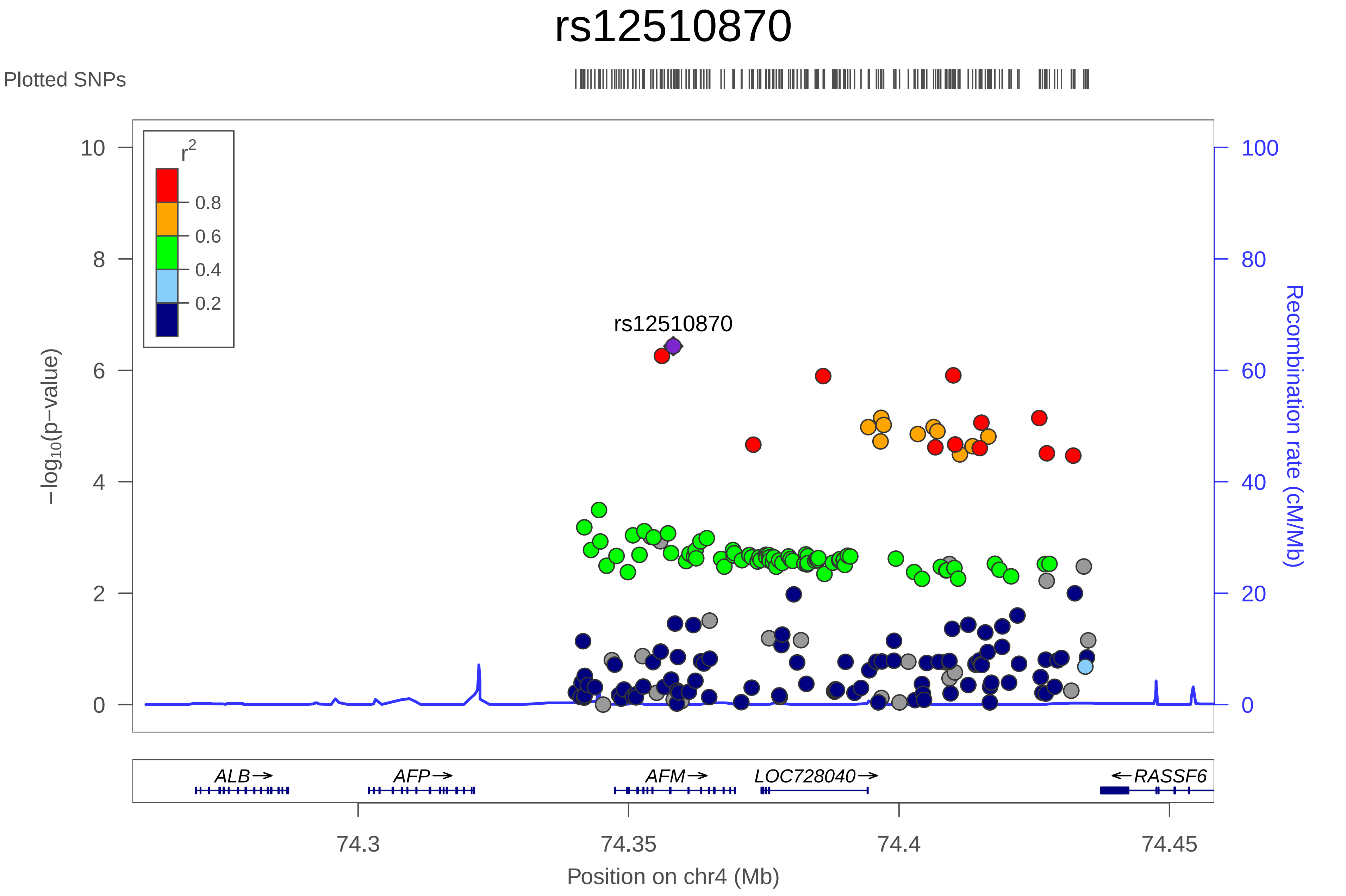

rs12510870
Plotted SNPs
r2
0.8
0.6
0.4
0.2
rs12510870
−log10(p−value)
Recombination rate (cM/Mb)
10
8
6
4
2
0
100
80
60
40
20
0
ALB→
AFP→
AFM→
LOC728040→
←RASSF6
74.3
74.35
74.4
74.45
Position on chr4 (Mb)

date:	Fri Jul 28 04:46:41 2017
build:	hg19
display range:	chr4:74258277−74458277 [74258277−74458277]
hilite range:	0 − 0 [0 − 0]
reference SNP:	chr4:74358277
number of SNPs plotted:	217
min P.value:	3.68E−7 [chr4:74358277]
max P.value:	9.98E−1 [chr4:74345293]

Make more plots at http://csg.sph.umich.edu/locuszoom/

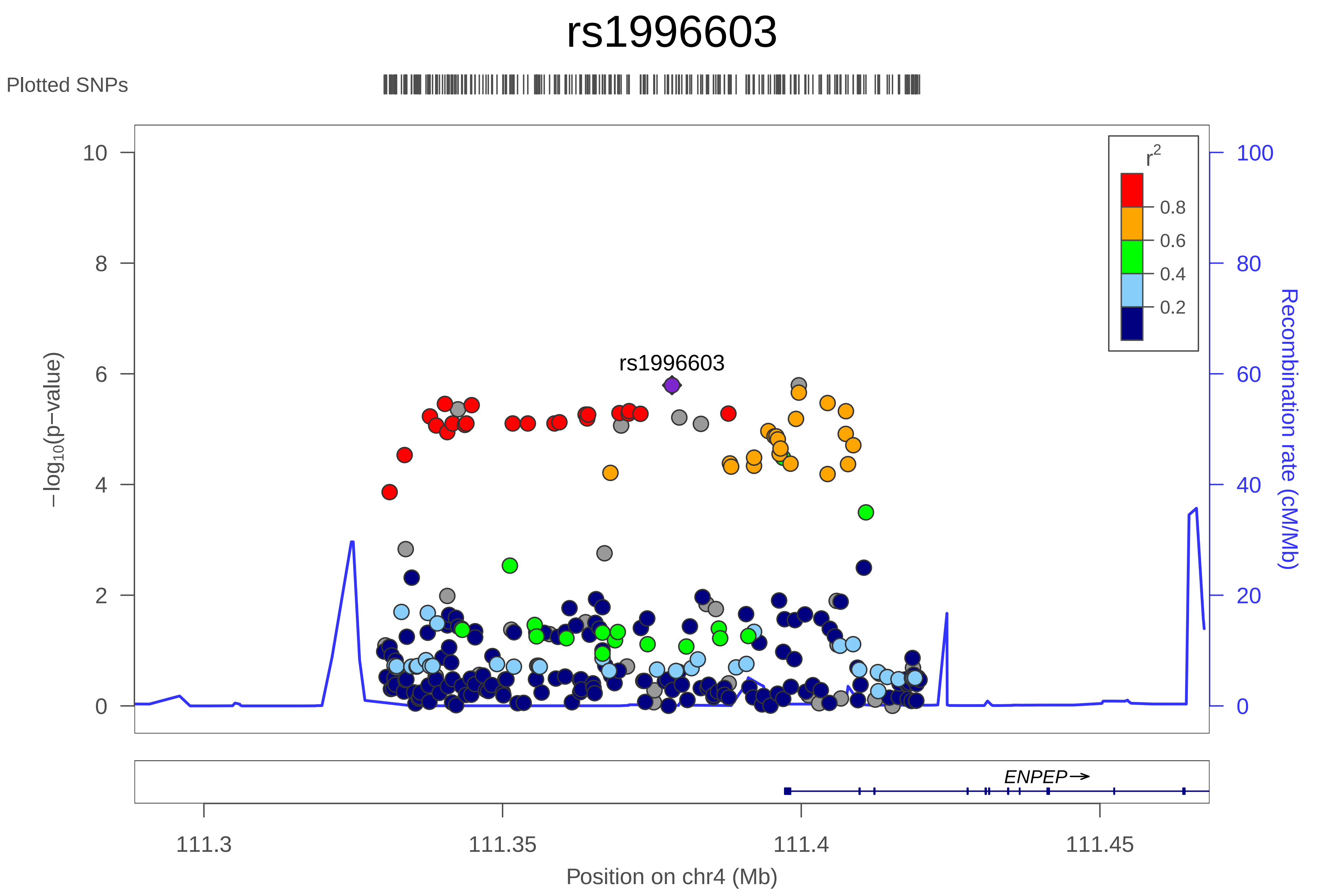
rs1996603
Plotted SNPs
−log10(p−value)
Recombination rate (cM/Mb)
r2
0.8
0.6
0.4
0.2
rs1996603
ENPEP→
111.3
111.35
111.4
111.45
Position on chr4 (Mb)

date:	Fri Jul 28 02:27:45 2017
build:	hg19
display range:	chr4:111288362−111468362 [111288362−111468362]
hilite range:	0 − 0 [0 − 0]
reference SNP:	chr4:111378362
number of SNPs plotted:	281
min P.value:	1.61E−6 [chr4:111378362]
max P.value:	9.96E−1 [chr4:111415274]

Make more plots at http://csg.sph.umich.edu/locuszoom/

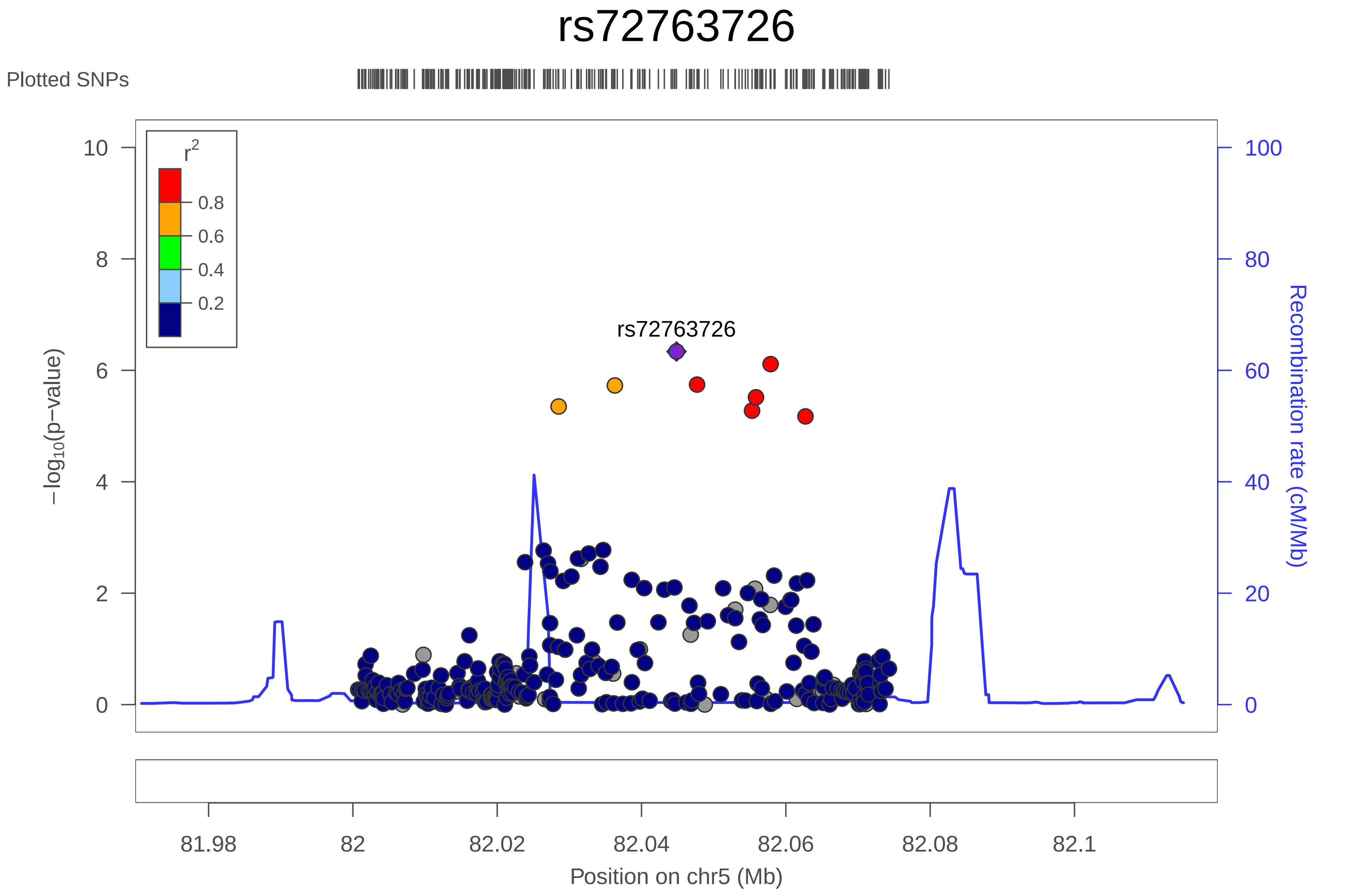
rs72763726
Plotted SNPs
r2
0.8
0.6
0.4
0.2
rs72763726
−log10(p−value)
Recombination rate (cM/Mb)
0
2
4
6
8
10
0
20
40
60
80
100
81.98
82
82.02
82.04
82.06
82.08
82.1
Position on chr5 (Mb)

date:	Fri Jul 28 04:51:17 2017
build:	hg19
display range:	chr5:81969846−82119846 [81969846−82119846]
hilite range:	0 − 0 [0 − 0]
reference SNP:	chr5:82044846
number of SNPs plotted:	296
min P.value:	4.6E−7 [chr5:82044846]
max P.value:	9.96E−1 [chr5:82012854]

Make more plots at http://csg.sph.umich.edu/locuszoom/

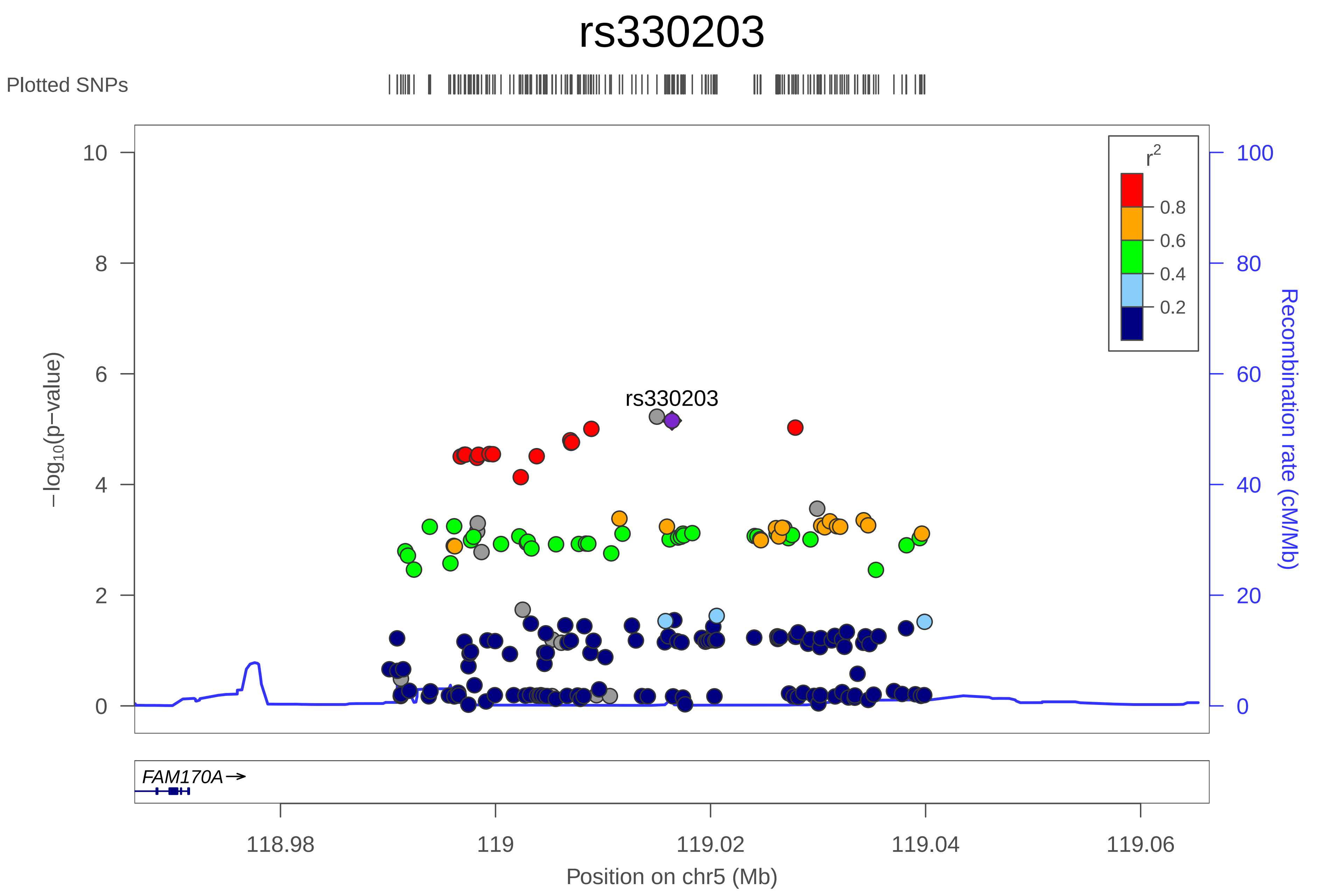
rs330203
Plotted SNPs
10
8
6
4
2
0
−log10(p−value)
100
80
60
40
20
0
Recombination rate (cM/Mb)
r2
0.8
0.6
0.4
0.2
rs330203
FAM170A→
118.98
119
119.02
119.04
119.06
Position on chr5 (Mb)

date:	Fri Jul 28 01:03:38 2017
build:	hg19
display range:	chr5:118966414−119066414 [118966414−119066414]
hilite range:	0 − 0 [0 − 0]
reference SNP:	chr5:119016414
number of SNPs plotted:	198
min P.value:	5.94E−6 [chr5:119015012]
max P.value:	9.51E−1 [chr5:118997478]

Make more plots at http://csg.sph.umich.edu/locuszoom/

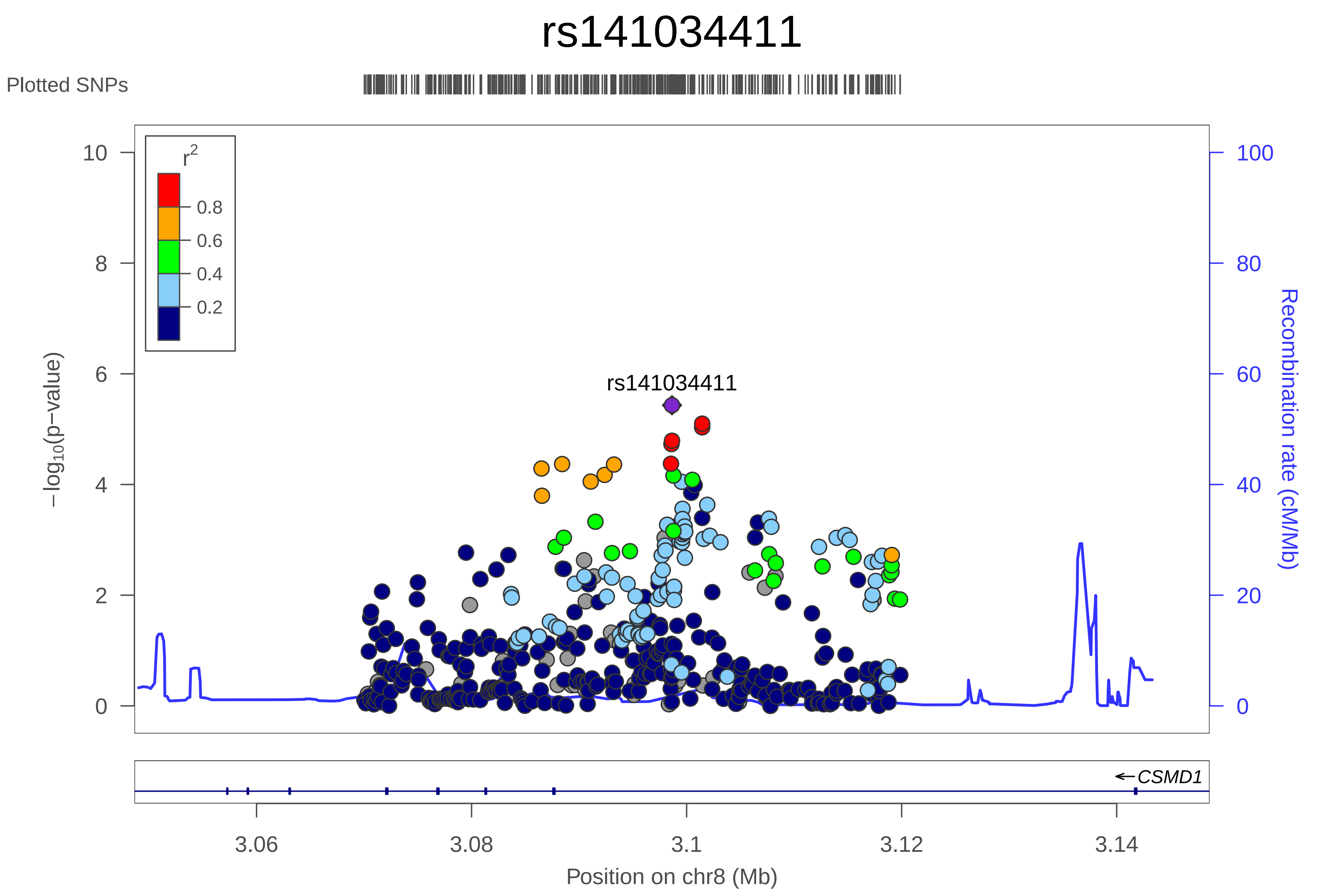

rs141034411
Plotted SNPs
r2
0.8
0.6
0.4
0.2
rs141034411
−log10(p−value)
Recombination rate (cM/Mb)
0
2
4
6
8
10
0
20
40
60
80
100
CSMD1
3.06
3.08
3.1
3.12
3.14
Position on chr8 (Mb)

date:	Fri Jul 28 04:58:58 2017
build:	hg19
display range:	chr8:3048640−3148640 [3048640−3148640]
hilite range:	0 − 0 [0 − 0]
reference SNP:	chr8:3098640
number of SNPs plotted:	459
min P.value:	3.69E−6 [chr8:3098640]
max P.value:	9.98E−1 [chr8:3107789]

Make more plots at http://csg.sph.umich.edu/locuszoom/

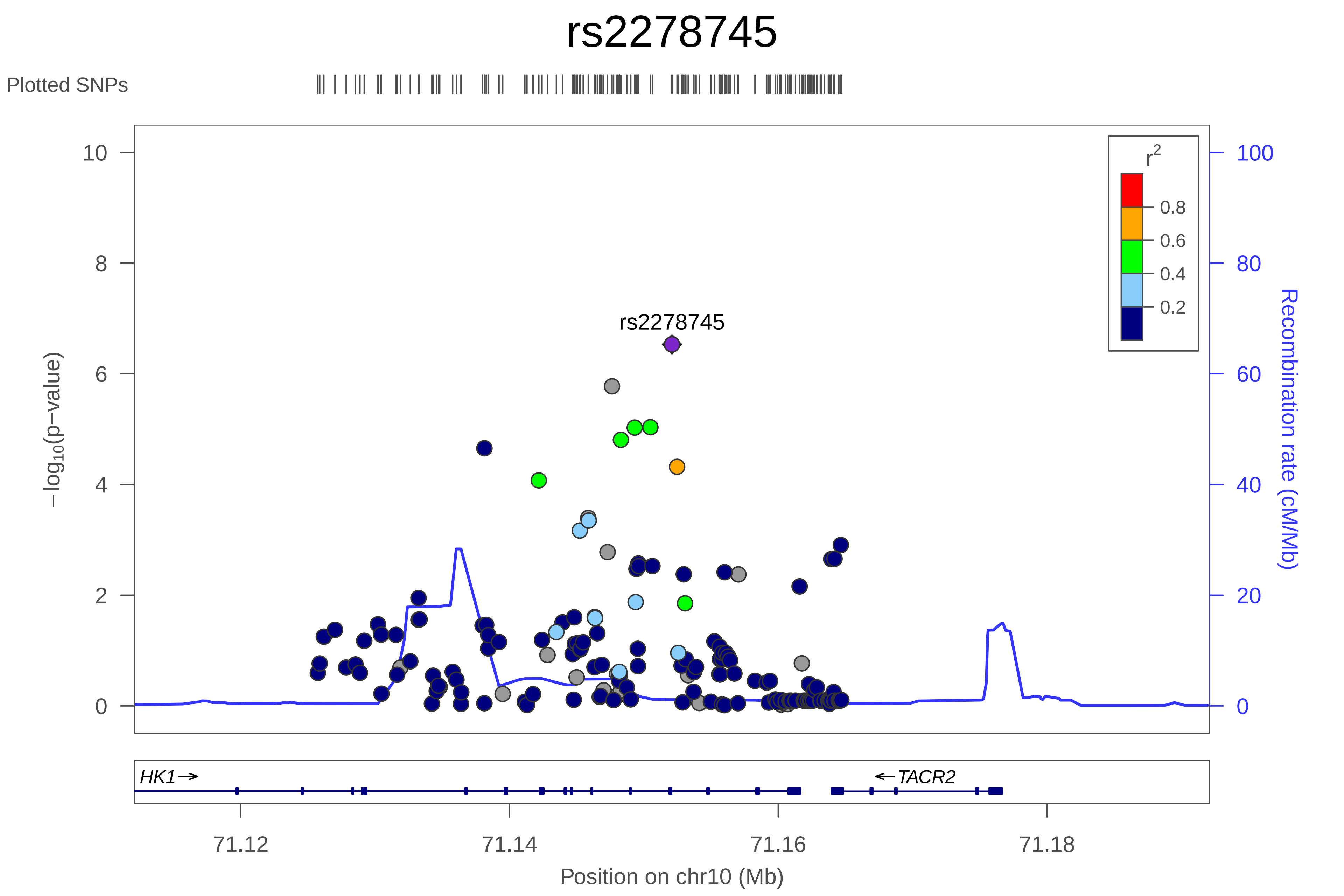
rs2278745
Plotted SNPs
rs2278745
r2
0.8
0.6
0.4
0.2
10
8
6
4
2
0
100
80
60
40
20
0
−log10(p−value)
Recombination rate (cM/Mb)
HK1→
←TACR2
71.12
71.14
71.16
71.18
Position on chr10 (Mb)

date:	Fri Jul 28 04:43:08 2017
build:	hg19
display range:	chr10:71112091−71192091 [71112091−71192091]
hilite range:	0 − 0 [0 − 0]
reference SNP:	chr10:71152091
number of SNPs plotted:	162
min P.value:	2.95E−7 [chr10:71152091]
max P.value:	9.7E−1 [chr10:71156005]

Make more plots at http://csg.sph.umich.edu/locuszoom/

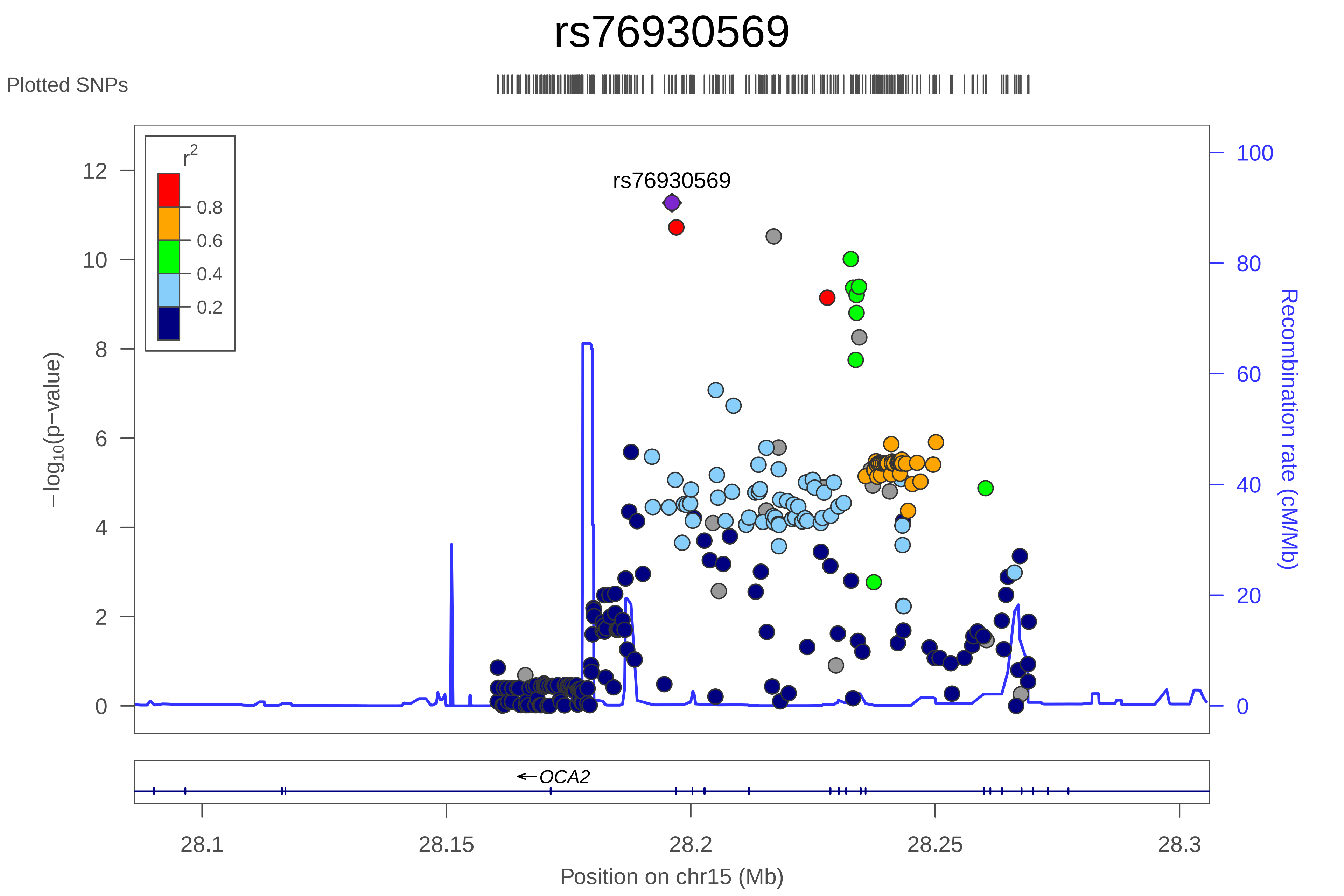
rs76930569
Plotted SNPs
r2
0.8
0.6
0.4
0.2
rs76930569
12
10
8
6
4
2
0
−log10(p−value)
100
80
60
40
20
0
Recombination rate (cM/Mb)
OCA2
28.1
28.15
28.2
28.25
28.3
Position on chr15 (Mb)

date:	Fri Jul 28 04:55:42 2017
build:	hg19
display range:	chr15:28086145−28306145 [28086145−28306145]
hilite range:	0 − 0 [0 − 0]
reference SNP:	chr15:28196145
number of SNPs plotted:	268
min P.value:	5.3E−12 [chr15:28196145]
max P.value:	10E−1 [chr15:28170694]

Make more plots at http://csg.sph.umich.edu/locuszoom/
