# Supplementary material for: Genome-wide association study of pigmentary traits (skin and iris color) in individuals of East Asian ancestry
Source: PeerJ. 2017 Nov 2;5:e3951. doi: 10.7717/peerj.3951 (PMC5671666; doi:10.7717/peerj.3951)
Supplement: Figure S11 — All these regions harbour multiple markers showing suggestive significance and good imputation scores (e.g., score info > 0.8). [file peerj-05-3951-s011.pdf]

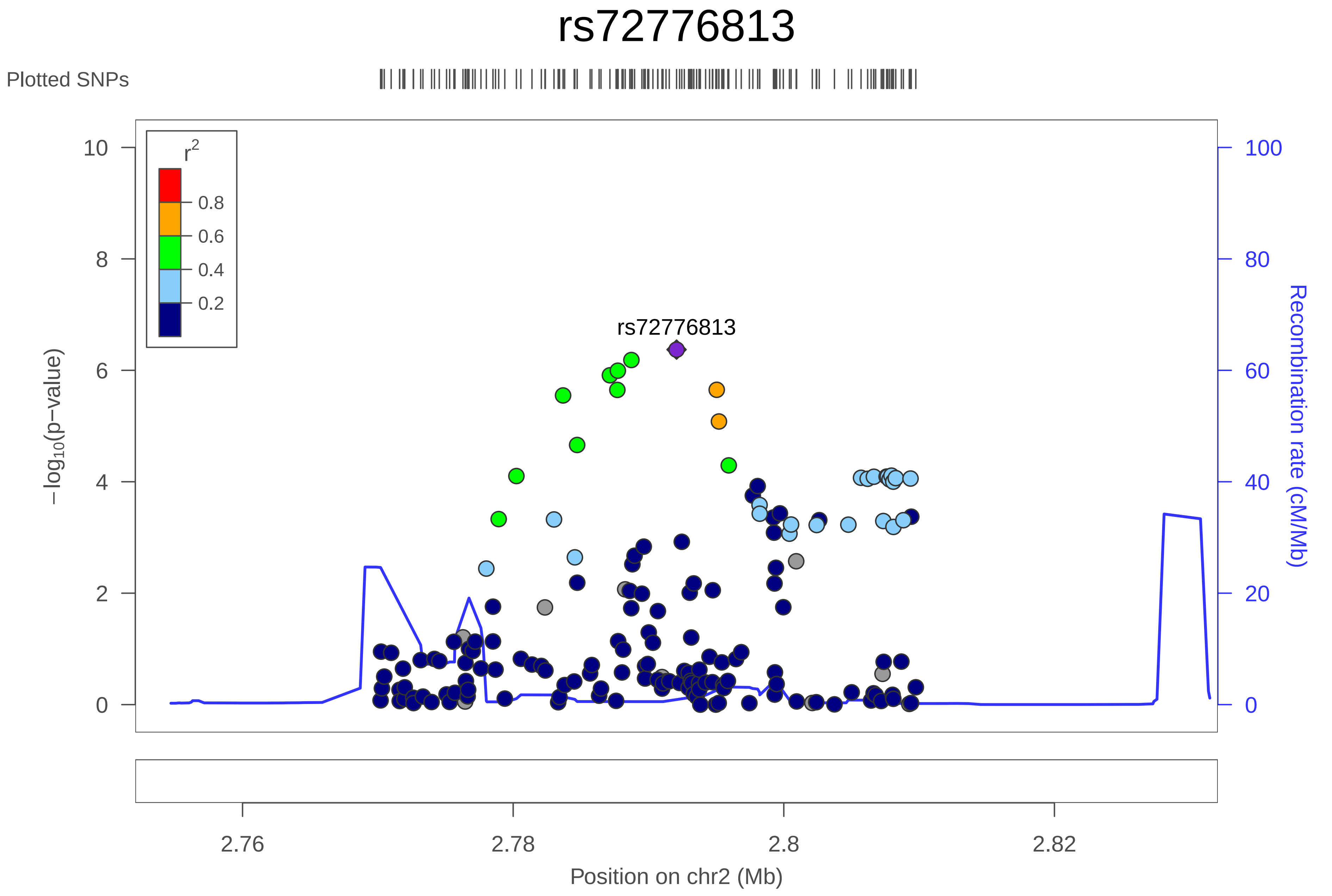
rs72776813
Plotted SNPs
r2
0.8
0.6
0.4
0.2
rs72776813
−log10(p−value)
Recombination rate (cM/Mb)
0
2
4
6
8
10
0
20
40
60
80
100
2.76
2.78
2.8
2.82
Position on chr2 (Mb)

date:	Fri Jul 28 05:39:29 2017
build:	hg19
display range:	chr2:2752075−2832075 [2752075−2832075]
hilite range:	0 − 0 [0 − 0]
reference SNP:	chr2:2792075
number of SNPs plotted:	168
min P.value:	4.25E−7 [chr2:2792075]
max P.value:	9.99E−1 [chr2:2793832]

Make more plots at http://csg.sph.umich.edu/locuszoom/

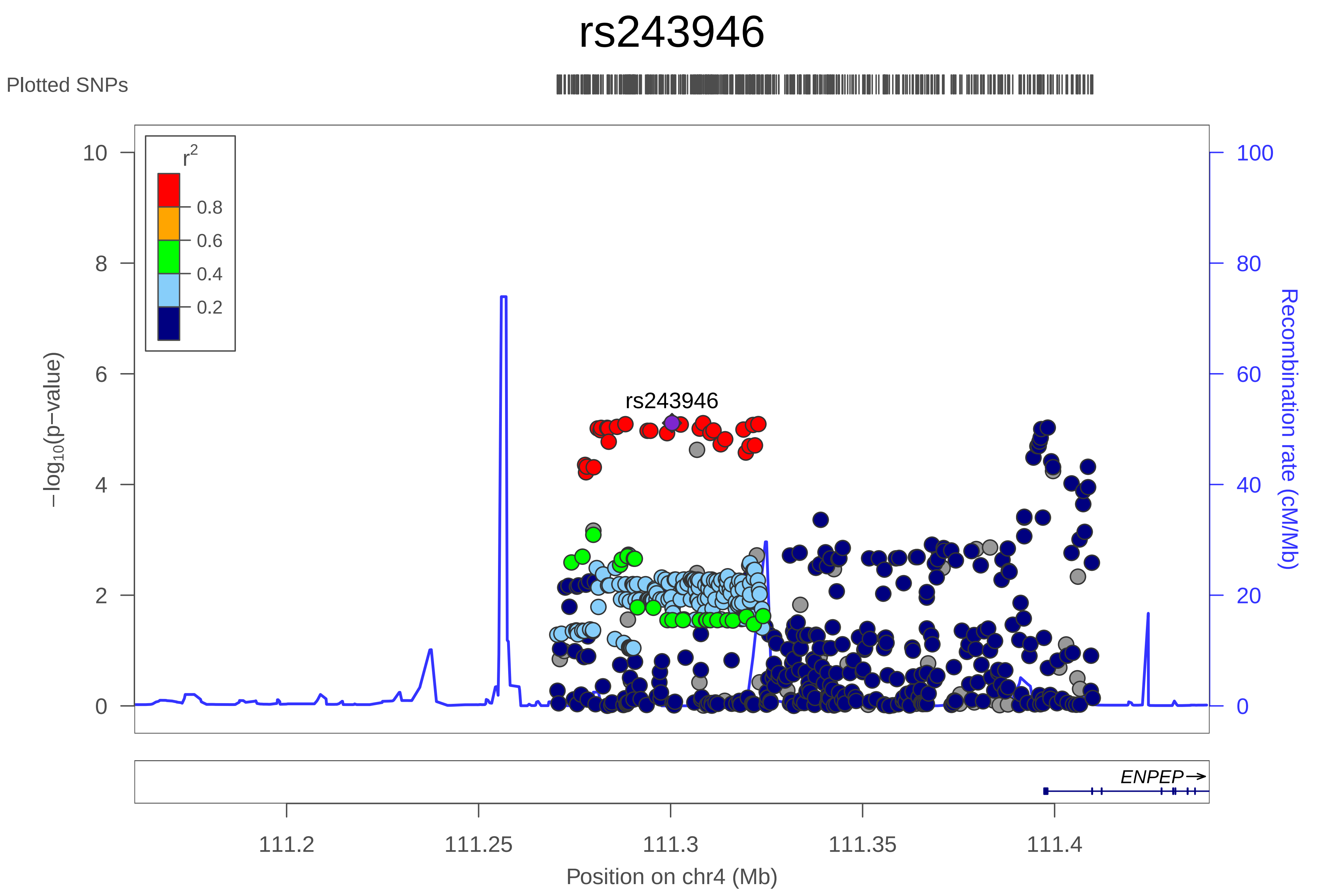
rs243946
Plotted SNPs
r2
0.8
0.6
0.4
0.2
rs243946
−log10(p−value)
Recombination rate (cM/Mb)
ENPEP
111.2
111.25
111.3
111.35
111.4
Position on chr4 (Mb)

date:	Fri Jul 28 05:11:02 2017
build:	hg19
display range:	chr4:111160362−111440362 [111160362−111440362]
hilite range:	0 − 0 [0 − 0]
reference SNP:	chr4:111300362
number of SNPs plotted:	562
min P.value:	7.74E−6 [chr4:111300362]
max P.value:	9.99E−1 [chr4:111356969]

Make more plots at http://csg.sph.umich.edu/locuszoom/

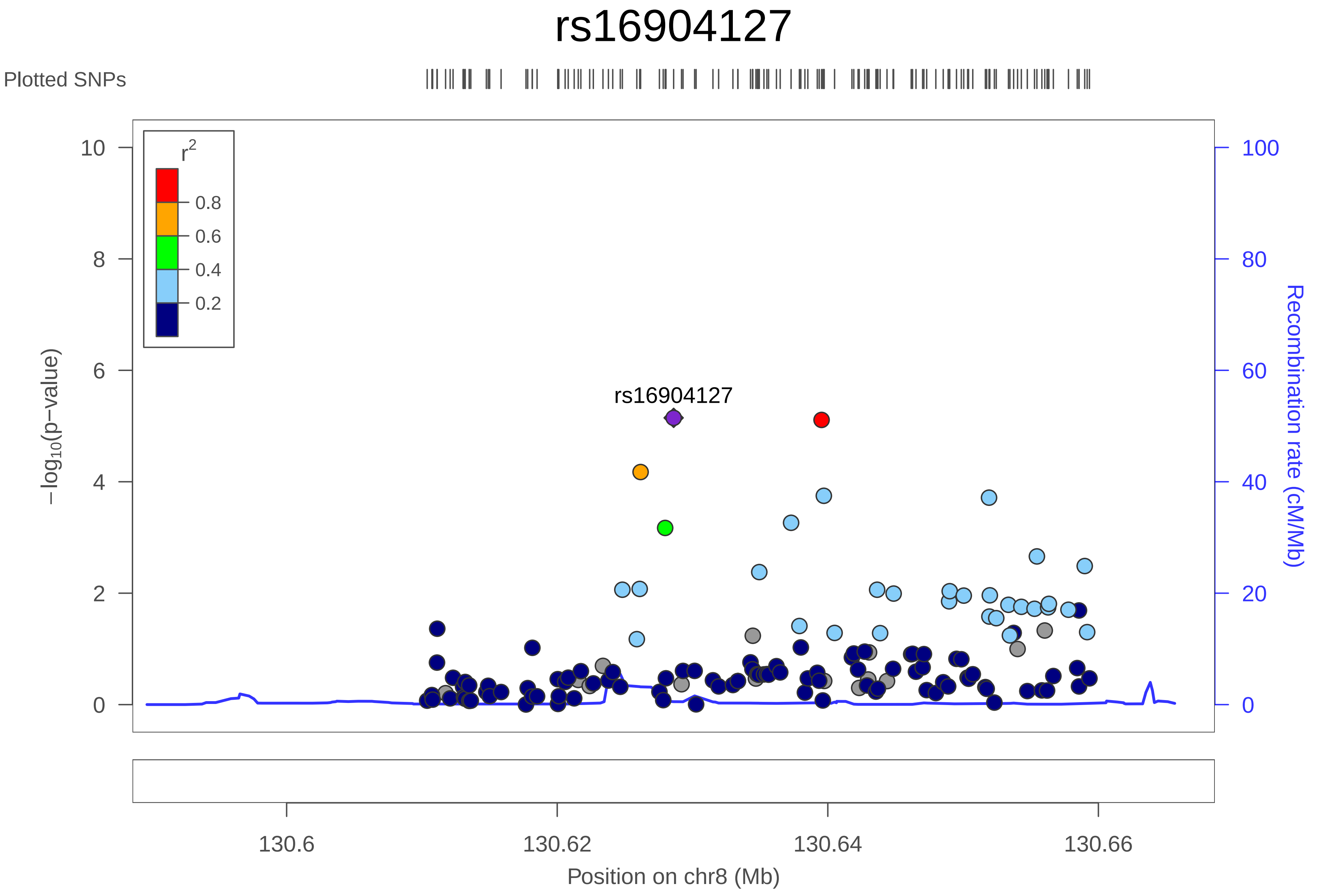
rs16904127
Plotted SNPs
r2
0.8
0.6
0.4
0.2
rs16904127
−log10(p−value)
10
8
6
4
2
0
100
80
60
40
20
0
Recombination rate (cM/Mb)
130.6
130.62
130.64
130.66
Position on chr8 (Mb)

date:	Fri Jul 28 05:43:18 2017
build:	hg19
display range:	chr8:130588606−130668606 [130588606−130668606]
hilite range:	0 − 0 [0 − 0]
reference SNP:	chr8:130628606
number of SNPs plotted:	143
min P.value:	7.13E−6 [chr8:130628606]
max P.value:	9.93E−1 [chr8:130617692]

Make more plots at http://csg.sph.umich.edu/locuszoom/

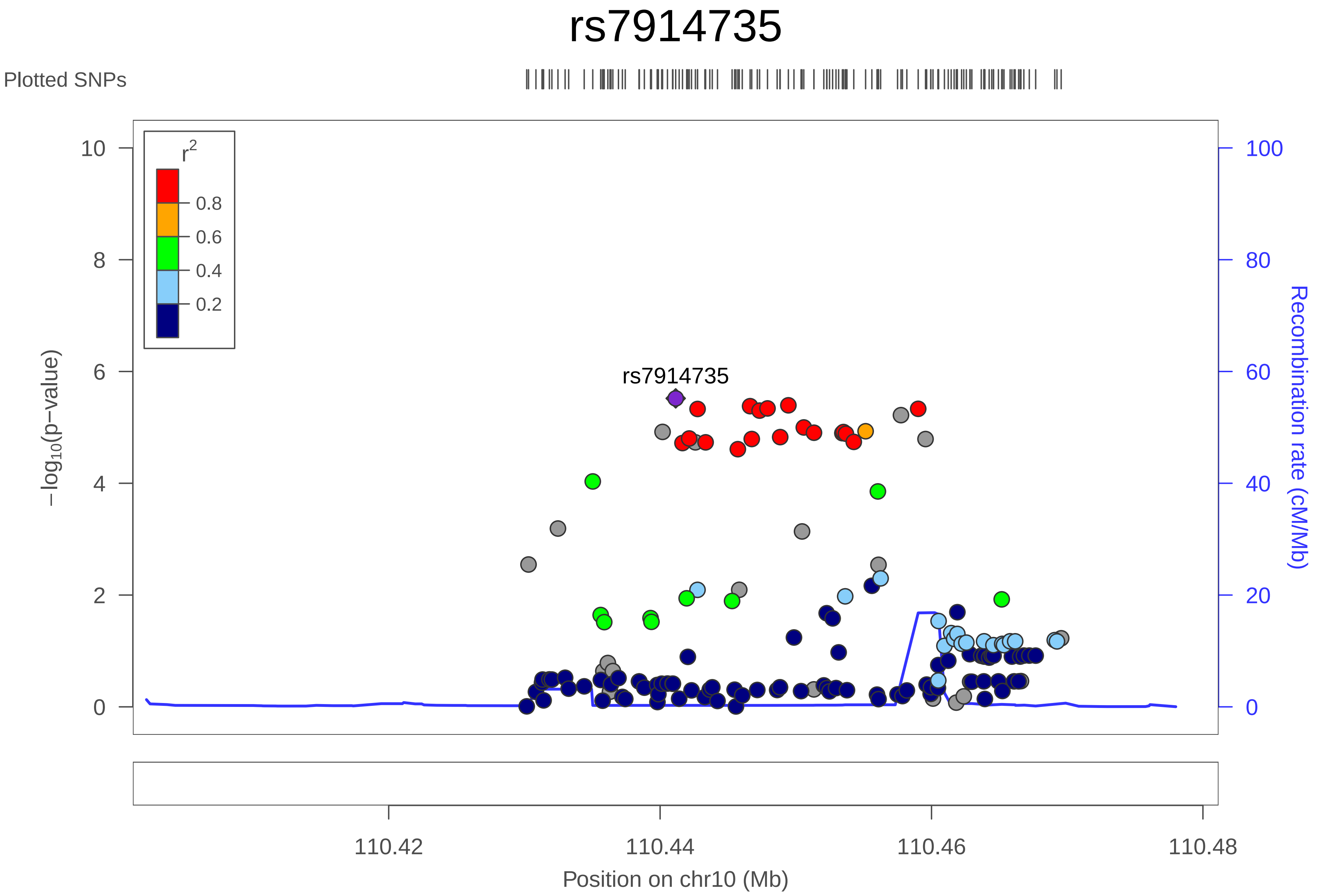

rs7914735
Plotted SNPs
r2
0.8
0.6
0.4
0.2
rs7914735
−log10(p−value)
Recombination rate (cM/Mb)
0
2
4
6
8
10
0
20
40
60
80
100
110.42
110.44
110.46
110.48
Position on chr10 (Mb)

date:	Fri Jul 28 05:22:06 2017
build:	hg19
display range:	chr10:110401149−110481149 [110401149−110481149]
hilite range:	0 − 0 [0 − 0]
reference SNP:	chr10:110441149
number of SNPs plotted:	151
min P.value:	3.03E−6 [chr10:110441149]
max P.value:	9.83E−1 [chr10:110445589]

Make more plots at http://csg.sph.umich.edu/locuszoom/

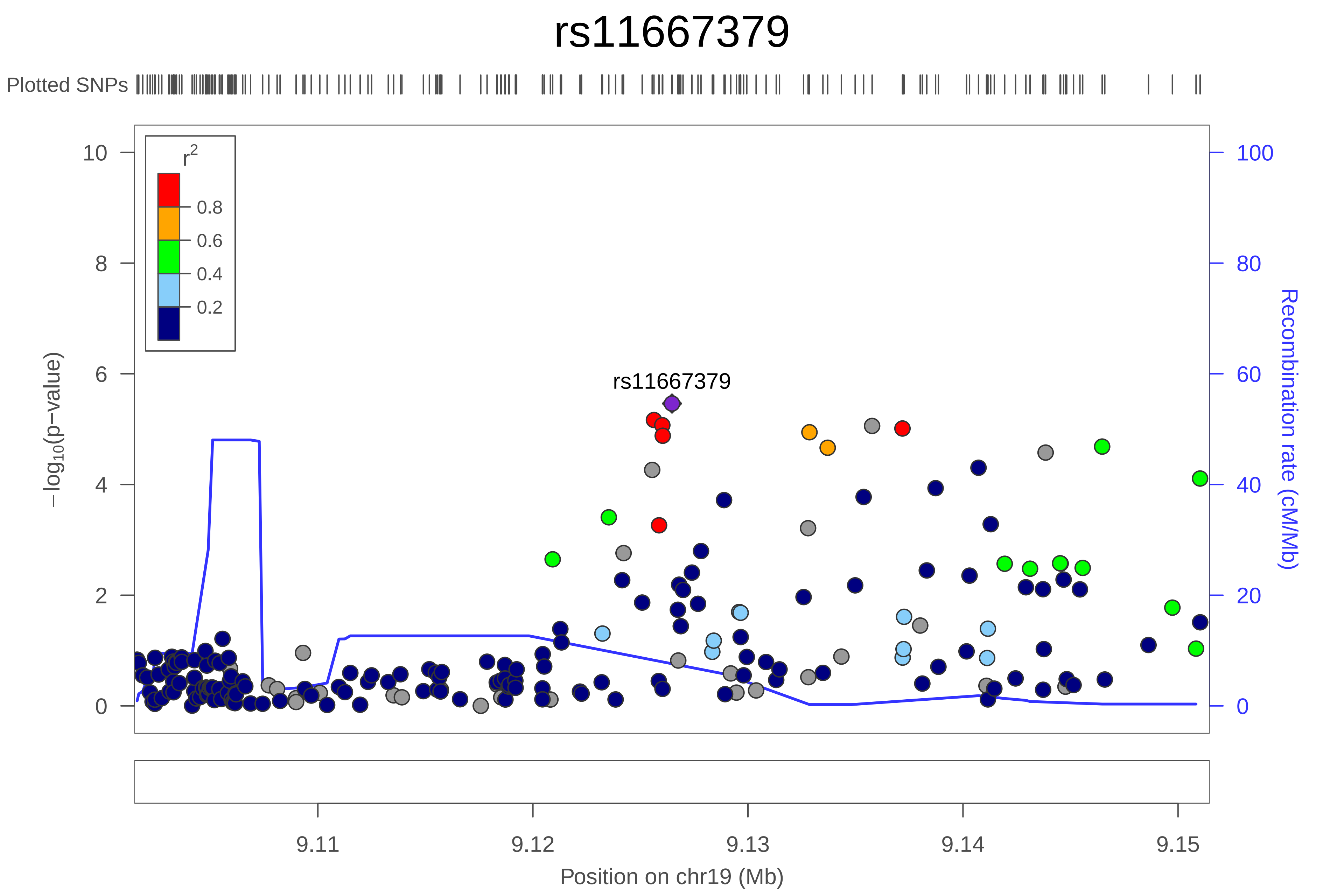

rs11667379
Plotted SNPs
r2
0.8
0.6
0.4
0.2
rs11667379
−log10(p−value)
Recombination rate (cM/Mb)
10
8
6
4
2
0
100
80
60
40
20
0
9.11
9.12
9.13
9.14
9.15
Position on chr19 (Mb)

date:	Mon Aug 7 13:51:19 2017
build:	hg19
display range:	chr19:9101468−9151468 [9101468−9151468]
hilite range:	0 − 0 [0 − 0]
reference SNP:	chr19:9126468
number of SNPs plotted:	205
min p−value:	3.45E−6 [chr19:9126468]
max p−value:	9.96E−1 [chr19:9117576]

Make more plots at http://csg.sph.umich.edu/locuszoom/
