# Supplementary material for: Genome-wide association study of pigmentary traits (skin and iris color) in individuals of East Asian ancestry
Source: PeerJ. 2017 Nov 2;5:e3951. doi: 10.7717/peerj.3951 (PMC5671666; doi:10.7717/peerj.3951)
Supplement: Table S1 — The table includes variants in the genes SLC24A5 and SLC45A2, which have very high frequencies in Europe and have been reported to have very strong effects on skin pigmentation in admixed groups. The table also includes variants that reached genome-wide significance in a recent GWAS of skin pigmentation in European samples (Liu et al., 2015). The effect allele is the allele that have been reported to decrease melanin levels in previous studies. [file peerj-05-3951-s012.docx]

Supplementary Table 1. Table reporting allele frequencies and effect size estimates in the East Asian sample for markers known to be associated with light skin in European populations. The table includes variants in the genes *SLC24A5* and *SLC45A2*, which have very high frequencies in Europe and have been reported to have very strong effects on skin pigmentation in admixed groups. The table also includes variants that reached genome-wide significance in a recent GWAS of skin pigmentation in European samples (Liu et al. 2015). The effect allele is the allele that have been reported to decrease melanin levels in previous studies.

| SNP | Gene | CHR | POS | NEA/EA | INFO | EAF-EAS | P-VALUE | BETA | SE | EAF-EUR^1^ |
| --- | --- | --- | --- | --- | --- | --- | --- | --- | --- | --- |
| rs16891982 | SLC45A2 | 5 | 33951693 | C/G | 0.712 | 0.021 | 0.922 | 0.091 | 0.937 | 0.938 |
| rs12203592 | IRF4 | 6 | 396321 | C/T | 0.501 | 0.024 | 0.576 | -0.585 | 1.045 | 0.116 |
| rs12913832 | HERC2 | 15 | 28365618 | A/G | 0.550 | 0.032 | 0.463 | -0.618 | 0.840 | 0.636 |
| rs1426654 | SLC24A5 | 15 | 48426484 | G/A | 0.730 | 0.016 | 0.017 | -2.460 | 1.029 | 0.997 |
| rs4268748 | MC1R | 16 | 90026512 | T/C | 0.983 | 0.274 | 0.898 | 0.034 | 0.265 | 0.228 |
| rs6059655 | RALY/ASIP | 20 | 32665748 | G/A | NA | NA | NA | NA | NA | 0.042 |
